# Three-dimensional (3D) liver cell models - a tool for bridging the gap between animal studies and clinical trials when screening liver accumulation and toxicity of nanobiomaterials

**DOI:** 10.1007/s13346-022-01147-0

**Published:** 2022-05-04

**Authors:** Melissa Anne Tutty, Dania Movia, Adriele Prina-Mello

**Affiliations:** 1 Nanomedicine and Molecular Imaging Group, School of Medicine, Trinity Translational Medicine Institute (TTMI), Trinity College Dublin, Dublin 8, Ireland; 2 Laboratory for Biological Characterisation of Advanced Materials (LBCAM), School of Medicine, TTMI, Trinity College Dublin, Dublin 8, Ireland; 3 Trinity St James’s Cancer Institute, Trinity College Dublin, St James’s Hospital, Dublin 8, Ireland

**Keywords:** Hepatotoxicity, 3D models, Nanobiomaterials, Liver, Nanomedicine

## Abstract

Despite the exciting properties and wide-reaching applications of nanobiomaterials (NBMs) in human health and medicine, their translation from bench to bedside is slow, with a predominant issue being liver accumulation and toxicity following systemic administration. In vitro 2D cell-based assays and in vivo testing are the most popular and widely used methods for assessing liver toxicity at pre-clinical stages; however, these fall short in predicting toxicity for NBMs. Focusing on in vitro and in vivo assessment, the accurate prediction of human-specific hepatotoxicity is still a significant challenge to researchers. This review describes the relationship between NBMs and the liver, and the methods for assessing toxicity, focusing on the limitations they bring in the assessment of NBM hepatotoxicity as one of the reasons defining the poor translation for NBMs. We will then present some of the most recent advances towards the development of more biologically relevant in vitro liver methods based on tissue-mimetic 3D cell models and how these could facilitate the translation of NBMs going forward. Finally, we also discuss the low public acceptance and limited uptake of tissue-mimetic 3D models in pre-clinical assessment, despite the demonstrated technical and ethical advantages associated with them.

**Graphical abstract:**

3D culture models for use as in vitro alternatives to traditional methods and conventional in vivo animal testing for testing liver accumulation and toxicity of nanobiomaterials

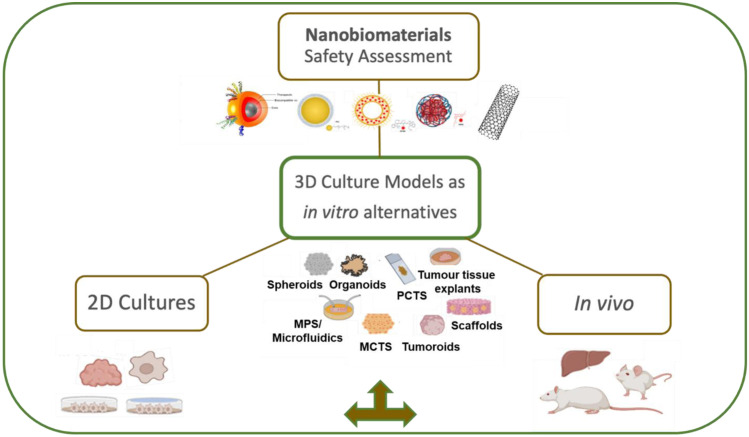

## Introduction

The many applications of nanobiomaterials (NBMs) have made them extremely beneficial in several fields today, most notably medicine. Regarding their medical applications, whilst NBMs offer many exciting new opportunities, their translation to the clinic is still slow. Up to recent years, the large investments into nanomedicine research, in fact, have only yielded a relatively small number of products that were successfully translated into clinical use to date [1]. This did shift with the development of two new mRNA vaccines for treating COVID-19 [2]; however, outside the area of vaccine development, progress is still slow and constitutes one of the main caveats of the nanomedicine research field [3]. The high attrition rate of NBMs is partly due to the insufficient and ineffective pre-clinical screening methods that are currently used for testing their toxicity in the body [4, 5], with one of the most common reasons for the withdrawal of nanomedicine products from the clinical market being NBM-induced liver injury [6], a factor which is often associated to a considerable liver-specific accumulation [7].

Structurally, the liver is highly vascularised, with its primary function to sequester and remove foreign materials from the body, including viruses, bacteria, and, indeed, NBMs. Its structure is well adapted for this purpose, with fenestrations in endothelial cells trapping foreign materials. Thus, the anatomy of the liver explains why non-specific liver accumulation and unintended hepatotoxicity are major obstacles in the clinical translation of NBMs. The vast majority of NBMs are in fact administered intravenously, where the liver constitutes the first pass metabolism. For example, Doxil^®^, the liposomal formulation of doxorubicin currently used in the clinic for many indications including breast and bladder cancer, acute lymphocytic leukemia, and Kaposi’s sarcoma, is metabolised mainly via the liver before being eliminated primarily via the biliary system. The consequence is that, whilst Doxil^®^ does reduce some of the negative side effects associated with free doxorubicin, such as cumulative and dose-dependent cardiotoxicity and neutropenic enterocolitis [8, 9], it still causes hepatic necrosis [10], among other issues.

From the considerations reported above, it is evident that the liver function is critical in defining both NBM safety profile and their ADME (absorption, distribution, metabolism, and excretion) once administered to humans. The use of sensitive and human-relevant liver models is therefore vitally important for increasing success with regard to clinical translation of NMBs.

In this context, this review presents the current state of the art in the pre-clinical assessment of NBMs, the limitations of current in vitro and in vivo liver models, and how tissue-mimetic 3D cell culture models can overcome such limitations, assisting in the translation of NBMs to the clinic. Finally, our review offers an insight into the potential reasons determining the limited adoption of these advanced 3D liver models along the pre-clinical R&D pipeline.

## Current state of the art in the pre-clinical assessment of NBMs

**Fig. 1:**
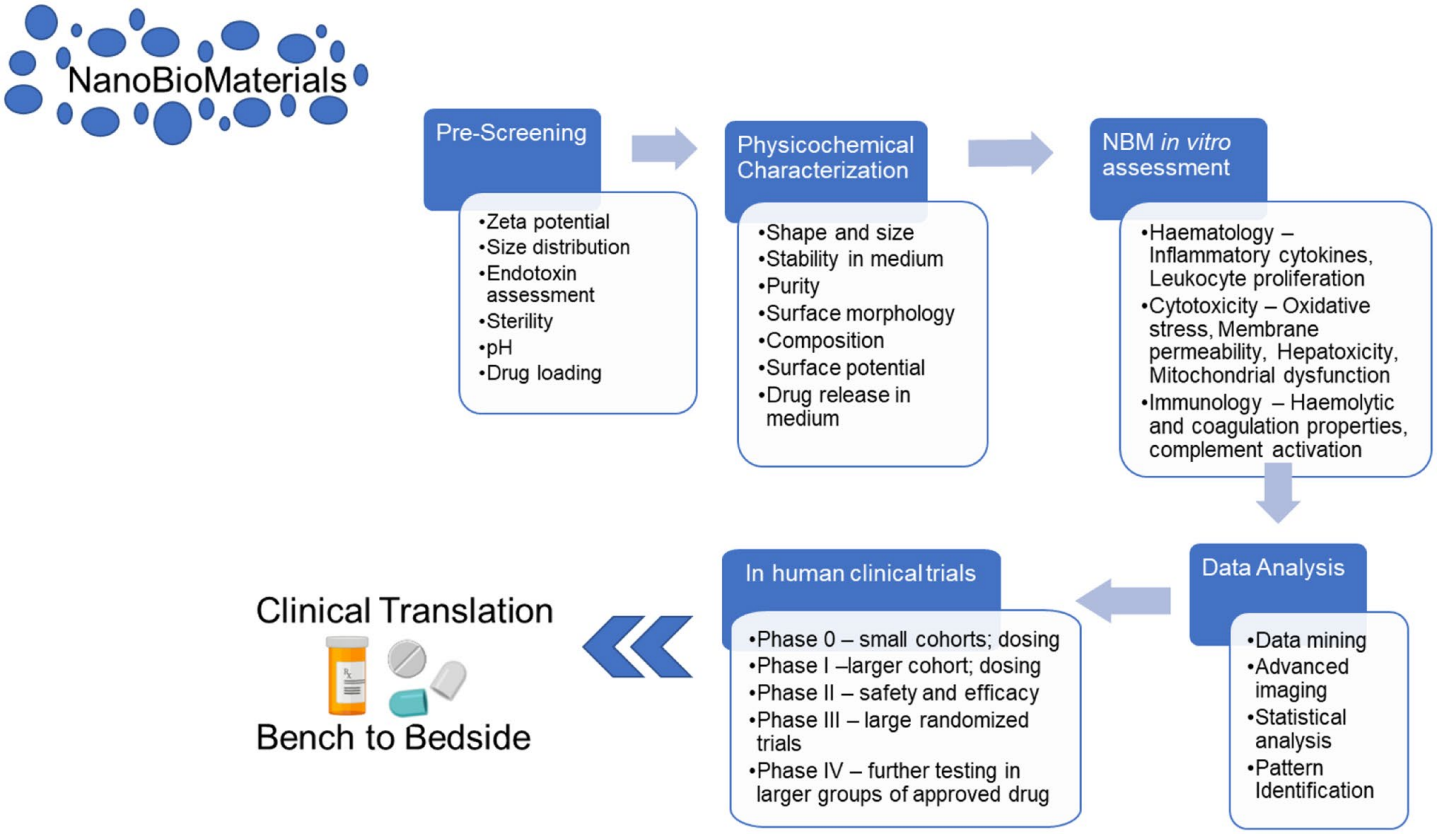
The pre-clinical assessment cascade for assessing NBM safety and efficacy. Following pre-screening and sterility assessment, a candidate NBM passes through physicochemical characterization, in vitro and in vivo experiments, before being deemed safe to enter human clinical trials

Since the approval of Doxil^®^/Caelyx^®^ in 1995 by the Food and Drug Administration (FDA), there has been a yearly increase in the number of biomedical applications for engineered NBMs [11–13]. As for conventional molecular drugs, several in vitro and in vivo pre-clinical tests (listed in Fig. 1) are carried out on NBMs prior to starting human trials. Briefly, following pre-screening (including sterility assessment), NBMs enter an assessment cascade covering areas such as physicochemical characterization, in vitro tests (e.g., haematology, cytotoxicity, and immunology), and in vivo tests (pharmacokinetics, biodistribution, and accumulation).

Focusing on the in vitro and in vivo assessment, the accurate prediction of human-specific hepatotoxicity is still a significant challenge to researchers [14]. Currently, in vitro two-dimensional (2D) cultures and in vivo testing in small mammals are the gold standards for determining acute hepatotoxicity of NBMs prior to clinical trials [15, 16]. Whilst conventional 2D in vitro cell-based models of the liver are extremely useful as first step in hepatotoxicity testing due to their low cost and ease of use, many limitations reduce their predictive power [17], namely the simplicity or the distinct lack of functional cross-talk between the cells forming the model in vitro [18], their poor ability to replicate the in vivo liver-like phenotypic properties, and liver physiology [19]. Similarly, in vivo animal models, despite being a key element of the regulatory requirements for both drugs and NBM pre-clinical assessment, are often unable to accurately predict human liver toxicity, due to the fundamental interspecies differences in both organ physiology and NBM uptake, degradation, and metabolism [20]. In recent years, scientific efforts focused on the development of new, more “human-relevant” technologies for determining the interactions and potential toxic effects of NMBs in the human liver.

## Human hepatic physiology and the importance of the liver regarding NBM

From production of bile to metabolising a large array of compounds, including NBMs, the liver plays many important roles in the human body. As an organ, the liver is the largest in humans, and has a diverse and varied cellular composition containing hepatocytes, parenchymal cells which comprise much of the liver at approximately 80% of total liver mass, and non-parenchymal cells (20% liver mass), cells which play roles in liver growth with respect to both their own proliferation and the proliferation of hepatocytes, in the form of Kupffer cells (KCs—liver resident macrophages), liver sinusoidal endothelial cells (LSECs–specialised endothelial cells), hepatic stellate cells (HSCs–pericytes which are the main effectors in fibrosis), fibroblasts, biliary epithelial cells, and various other immune cells and adult stem cells [21].

**Fig. 2:**
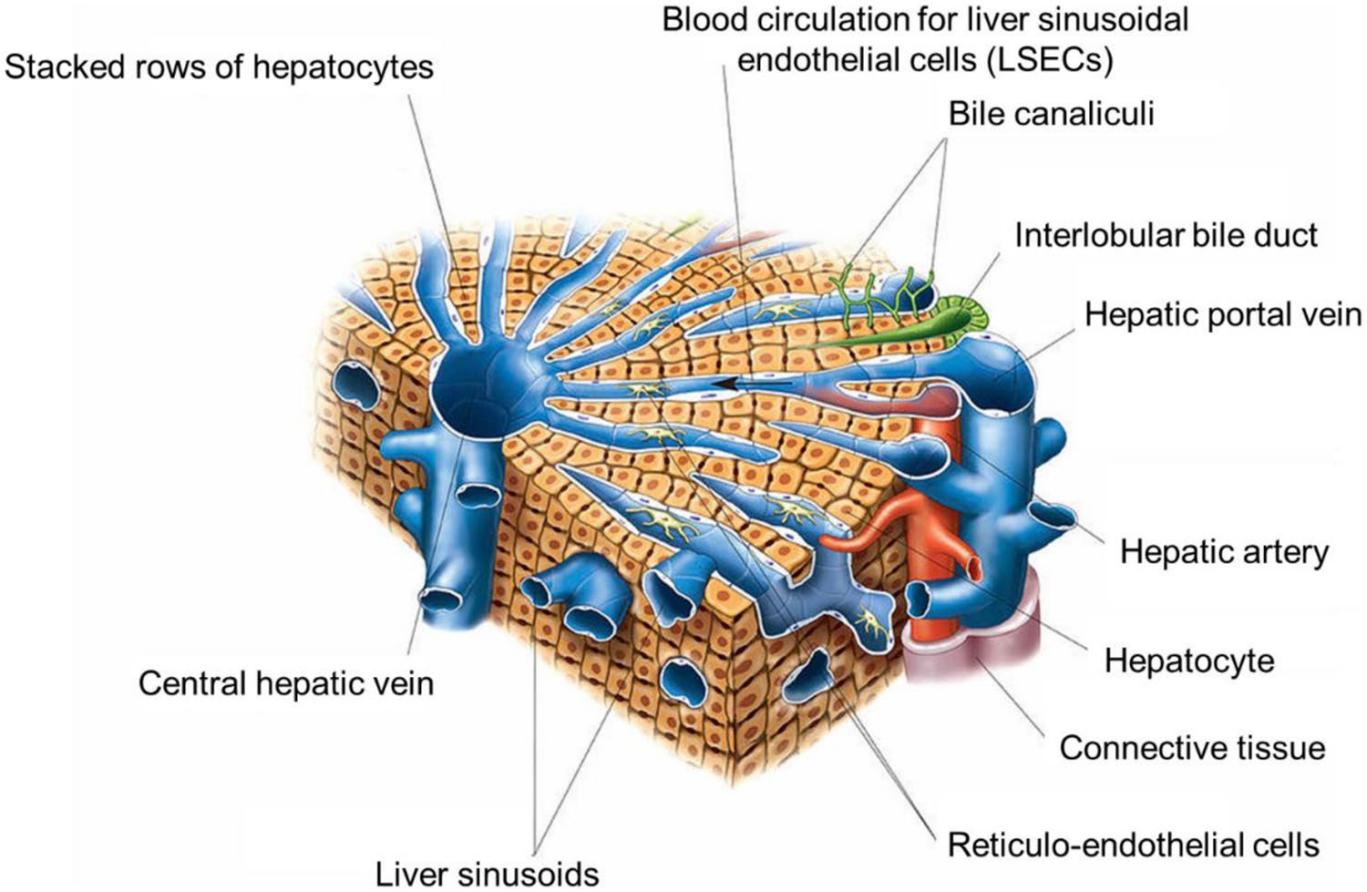
Structure of the lobule of the liver. This illustration includes many key elements of liver phenotype and function that cannot be successfully replicated in 2D culture, including ordered stacking of hepatocytes, formation of bile canaliculi, and blood circulation. Adapted from “Asklepios Atlas of the Human Anatomy” [22]

As an organ, the human liver is complex and highly vascularised (as observed in Fig. 2). Connected by the hepatic artery carrying blood from the aorta, and by the portal vein carrying blood from the gastrointestinal tract (GIT), pancreas, and spleen, the liver also has a heterogenous cellular composition, incorporating hepatocytes (the primary target of disease and the most abundant liver cell in terms of both volume and quantity), the liver resident macrophages known as Kupffer cells, hepatic stellate cells (HSCs), fibroblasts, immune cells, biliary epithelial cells, and adult stem cells [18]. Another essential component of the liver is the cytochrome P450 (CYP450) family of enzymes, vitally important to liver function as they mediate drug and NBM metabolism [18]. Each lobe of the liver comprises approximately one million lobules, around 1 mm × 2 mm in size, organised in a hexagonal manner around the central vein. This structure leads to lobule zonation, whereby zone 1 is the periportal zone, closest to the vasculature and most densely supplied with oxygen, nutrients, and blood (non-parenchymal cells including hepatic stellate cells and bile duct cells are also more abundant in this zone); zone 2 is the transitional region between zone 1 and 3; and zone 3, the perivenous zone, is nearest the central vein and less densely supplied with oxygen, blood, and essential nutrients [23, 24]. It is this formation that leads to liver functional zonation, whereby hepatocytes in different zones exhibit different functionality. It is vitally important that liver zonation is considered when undertaking liver modelling, as zonation is disrupted in diseases, particularly the ones associated with reactive oxygen species and hypoxia, such as hepatocellular carcinoma (HCC) and non-alcoholic fatty liver disease (NAFLD) [18]. Bile canaliculi, another essential liver component, collect bile formed in the liver, which in turn is drained in bile ducts before entering either the gall bladder or duodenum [25].

### Interaction of NBMs with the liver

The liver is a key organ of interest for NBM toxicity responses, for a variety of different reasons [26, 27]. Approximately 30–99% of NBMs accumulate and are sequestered in the liver following administration, reducing the amount of NBMs reaching the target tissue and potentially leading to unintended hepatotoxicity. Thus, the interactions between NBMs and liver cells determine the fate of the NBM in vivo; however, to date, the specific combinations of physicochemical properties that determine sequestering to the liver remains unknown. Whilst in vivo studies mainly focus on NBM accumulation at the organ level, in vitro assessment focuses on a single hepatic cell type in culture, not considering the unique 3D arrangement and architecture, as well as cellular composition, of the liver and how these affect NBM interactions. For example, it is widely detailed in literature that many NBMs are taken up by NPCs despite these being the less abundant cell type in the organ, and that NBMs which are taken up by hepatocytes are largely cleared in the body by the hepatobiliary pathway. The interaction between NBMs and various specific liver cell types is discussed further below.

### Role of hepatocytes in the liver

The primary functioning cell type in the liver is the hepatocytes, cuboidal hepatic epithelial cells which line the sinusoids. They sit in plates along the hepatic lobule, between systems of capillary sinusoids that connect the portal tracts to the central vein. The central vein facilitates a consistent supply of blood and other materials. Hepatocytes are tightly connected with each other to form cell plates via junctions, including gap, tight, and adherens junctions. Gap and tight junctions play a critical role in bile secretion, one of the most differentiated functions of the liver. Hepatocytes form canalicular-like structures, which run perpendicular to capillaries and are a key component of the liver structure and functionality [79]. Hepatocytes are also functionally polarised cells, which contain specific transporters localised to both the apical (or canalicular) or basolateral (sinusoidal) membranes. This polarity is vitally important for efficient liver functions, and it allows molecules/compounds to be taken up or effluxed into the bile or metabolised and transported back into the bloodstream [23, 24, 28]. Hepatocytes play many important roles with regard to metabolic function, production of bile, detoxification of materials, and protein synthesis [29], and they express a variety of plasma proteins, including protease inhibitors, transporters, inflammatory modulators, and albumin, and activate innate immunity as a defence mechanism against invading microorganisms by secreting innate immunity proteins [30, 31]. Regarding their interactions with NBMs, hepatocytes themselves endocytose NBMs, releasing them back in the bloodstream or into bile; however, to date, uptake of NBMs in hepatocytes has only been observed when very large doses of NBMs are administered or in instances where macrophages have been chemically depleted [32]. Various factors appear to influence NBM uptake by hepatocytes, including PEGylation and positive surface charges [33]. Size also plays a role, with uptake into hepatocytes normally occurring for NBMs below 50 nm [27]. Hepatobiliary clearance of NBMs occurs actively, promoted by various liver transporters and facilitated by a variety of drug-metabolizing enzymes, followed by either secretion into the bile duct via bile canaliculi or being filtered back into the bloodstream [34]. An understanding of hepatocyte targeting is critical to removing NBMs via the hepatobiliary route, and today there are various strategies for designing NBMs to both enter and interact with hepatocytes, harnessing either (1) transcytosis via the endothelial cell lining or (2) the sinusoidal intercellular junctions. As hepatocytes make up such a vast proportion of the cell of the liver, many NBMs have also been specifically designed to target these cells, by the active targeting of low-density lipoprotein (LDL), high-density lipoprotein (HDL), asialoglycoprotein (ASGP) and glycyrrhizin/glycyrrhetinic acid receptors, and the immunoglobulin A binding protein. A detailed overview of the interactions between hepatocytes and NBMs (and other hepatic cell types) can be found in a comprehensive review from Zhang et al. [27].

### Role of non-parenchymal cells in the liver

Liver non-parenchymal cells, or NPCs, make up for an approximate 20% of liver volume and NPCs play many roles in the liver, primarily in maintaining hepatic structure and functionality. Liver damage significantly alters both the phenotype and function of a non-parenchymal cell, significantly reducing the ability of the liver to recover appropriately. Three of the most abundant and important NPC of the liver are sinusoidal endothelial cells (LSECs), Kupffer cells, and stellate cells. Other important NPC types that also reside in the liver but are less abundant are fibroblasts, neutrophils, and macrophages. With regard to their interactions with NBMs, NPCs play vital roles, with NBMs interacting with varying NPCs, before reaching hepatocytes. Kupffer cells recognise NBMs as foreign materials, with NBMs being internalised through scavenger receptors and subsequently taken up by micropinocytosis, clathrin-mediated and caveolin-mediated endocytosis, and various other endocytosis pathways [35]. It is the Kupffer cells, along with the blood-circulating monocytes and macrophages in other tissues including spleen and gut, which constitute the mononuclear phagocyte system, or MPS. The MPS, also known as the reticular endothelial system, is responsible for sequestering approximately 95% of NBMs administered [36], with the rate of uptake highly dependent on NBM characteristics such as surface chemistry, size, and ligand chemistry. It is reported that larger NBMs, ranging in size from 400 to 600 nm, are preferentially phagocytosed by these cells, along with particles that are neutrally charged (i.e., PEG coatings). Studies have also shown that rod-shaped NBMs exhibit a reduced clearance compared to their spherical counterparts, potentially due to the presence of fewer accessible binding areas for interactions with macrophages [37, 38]. Various studies have highlighted this phenomenon, with Lunov et al. showing that 20-nm and 60-nm SPIONs accumulated irrespective of their size in macrophages via clathrin-mediated and scavenger receptor A endocytosis, with the larger SPIONs exhibiting up to 60 times greater uptake than the smaller ones [39]. Kupffer cells have also been shown to take up low-density lipoprotein (LDL) NBMs and liposomes of sizes ranging from 27 to 590 nm [40]. Despite their low abundance and challenging location, i.e., their residence in the space of Disse adjacent to endothelial cells, hepatic stellate cells (HSCs) have also been shown to take up various NBMs, including liposomes, AuNPs, SPIONS, and polymeric NBMs [41–45]. HSC-targeting NBMs also have implications in the treatment of liver fibrosis, with surface receptors allowing NBMs to directly target HSCs, provided they have not been removed from circulation by LSECs or Kupffer cells [27]. LSECs, the cell type which forms continuous linings along the vasculature of the liver sinusoid, may also have implications for NBMs, with studies from Kamps et al. Akhter et al. and Kren et al. illustrating that they can successfully take up both AuNPs, micelles and liposomes [40, 46, 47].

## Pre-existing methodologies for assessing hepatotoxicity

### 2D cultures from immortalised cell types

A significant challenge today is the accurate prediction of human-specific liver toxicity. It is widely reported that animal models often do not reflect human specific toxicity due to disease adaptations, interspecies variation, and fundamental differences in physiology. Comparably, in vitro models often do not predict toxicity accurately due to significant issues such as non-organ-specific toxicity, non-linear dose–toxicity, unclear mechanisms, and lack of the key structural and functional characteristics listed above, including lack of liver cell heterogeneity, zonation, formation of secondary structures, and in vivo-like cell density. In vitro liver models, in their two-dimensional biological configuration, have been long established; nonetheless, since the development of 3D biology, several issues have impacted their utilization, as summarised in Table 1. Given the complexity of the organ and its many roles involved, it is not strange to see that the main limitations for the 2D models reside on the composition and its flat configuration. In vitro 2D cell culture models are mainly monocultures formed from human immortalised or transformed cell lines, such as HepG2 [48–52], C3A [53, 54], Huh7 [48, 55, 56], and HepaRG [48, 56, 57] cells. Hepatocytes, the cuboidal hepatic epithelial cells that line the sinusoids, are the primary functioning cell type in the liver with their functions ranging from detoxification of toxic materials to basic metabolic functions [29]. They also activate innate immunity as a defence mechanism against the introduction of external materials such as NMBs [30]. Immortalised hepatic cell lines, normally derived from human hepatocellular carcinoma, have been the “go-to” for researchers for decades for a variety of reasons, including their wide availability from certified cell line sources such as ATCC (i.e., the American Type Culture Collection, which is a non-profit organisation that collects, stores, and distributes standard reference cell lines and other research and development materials), or BIOPREDICT (whose most popular product is the HepaRG cell line), relative ease of handling and culture, lack of inter-donor variation [48, 58–60], long-term maintenance in culture with a stable phenotype (depending on cell type), and resistance to senescence [61]. For decades, in vitro 2D hepatocyte models have been successfully used to obtain pre-clinical data on many NBMs, including the liposomal forms of irinotecan (including studies undertaken in SK-Hep-1 cells) [62] and doxorubicin (HepG2 cells, among others), gold [63–65], SPIONs [66], and carrier NBMs such as poly(alkyl cyanoacrylate) or PACA [67, 68] and poly(butyl cyanoacrylate) or PBCA.

**Table 1:** 2D hepatocyte culture models: advantages and disadvantages. Abbreviations: *CYP450*, cytochromes P450; *OECD*, Organisation for Economic Co-operation and Development.

**2D hepatocyte culture models**	**Advantages**	**Disadvantages**	**Reference**
**Monoculture**	Current gold standard for drug toxicity and metabolism studies	Low predictive value due to monocellular composition	[12, 22–24, 27–29, 31, 32]
	Easy and cost-effective	Inability to reproduce liver architecture	
		Rapid loss of cell morphology and polarity	
	Small set-up costs	Rapid loss of ability to metabolise drugs	
		Decreased albumin synthesis	
**Co-culture/multicellular**	Incorporation of multiple hepatic cell types means greater in vivo relevance and tissue-mimetic responses to inflammatory stimuli	Limitations with the number of cell types that can be co-cultured	[36, 45, 46, 48, 50, 51]
		Lack of extracellular matrix (ECM) components	
	Hepatocyte-specific morphology/phenotype is maintained	High intra-laboratory variability	
	Increased CYP450 enzyme induction/activity	No standard, OECD-approved co-culture testing models established	
	Stable albumin production for up to 37 days		
	Increased phase I and II enzyme expression	Optimization is needed to determine appropriate culture conditions	

Despite being widely used as early predictors of NBM liver toxicity in pre-clinical assessment [20, 69], on the other hand, cell lines used to form 2D cultures exhibit many disadvantages as presented in Table 1. Hepatic cell lines have reduced metabolic capacities, exhibit genomic content, and can have altered phenotypes, upregulated expression of inflammatory mediators, loss of cell polarity and contact inhibition, and, most notably, decrease in specific liver function, e.g., reduced CYP450 activity [70–73]. It has previously been shown that, to induce an increase in CYP enzyme activity in the HepaRG cell line, the cells must be treated with high concentrations of DMSO, a response which is artificial and counterproductive, as it alters the hepatotoxic responses to NBM exposure and, subsequently, skews data obtained from cytotoxicity assessment [48]. In the HepG2 cell line, the expression of phase I and II enzymes is also dramatically lower when compared to primary human hepatocytes (PHH); therefore, hepatotoxins are not as readily or accurately detected in immortalised HepG2 cell cultures as they would be in vivo.

### Primary human hepatocyte models

It is possible to overcome the major caveats of hepatocyte cell lines by using PHH, i.e., cells extracted from human liver biopsies [74–76]. When cultured in monolayers, the PHH metabolic capacity is comparable to in vivo hepatocytes, particularly with regard to albumin secretion, regulation of phase I and II metabolic pathways, expression of specific liver functional markers, uptake and metabolism, ammonia detoxification, and glucose metabolism [77]. This makes PHH the most accurate and physiologically relevant hepatic cell model to date [58]. In spite of this, PHH cultures are not without limitations. Supply of these cells is limited and protocols for extraction are complex. There is also an added issue of inter-donor variation and ability to maintain a stable phenotype, with wild-type characteristics only maintained for a limited time (up to 72 h) in 2D, due to de-differentiation mechanisms induced by the cell growth on flat culture surfaces, reducing the predictive power of the model [78]. Wild-type characteristics are also reduced from handling of the cells in culture, with changes in cell metabolism (e.g., reduction in CYP450 enzyme activity) and senescence observed at low passage numbers [74, 75]. A further issue is observed with cell seeding density, normally approximately 1% of physiological density, something which again impacts intercellular signalling and the predictive power of the model [79]. To avoid these problems, fresh PHH must be extracted from human biopsies regularly; however, this incurs in large research costs and dramatically increases the labour and time associated with liver toxicity screening of NBMs based on PHH models.

### 2D liver co-cultures

A further way of overcoming the issues detailed above with regard to the composition of conventional 2D liver cultures is by forming cultures comprising multiple liver cell types. The liver is an extremely diverse organ, with varied cellular composition containing not only hepatocytes, but also many non-parenchymal cell types (comprising the 20% of the total liver mass) in the form of Kupffer cells, liver sinusoidal endothelial cells (LSECs), hepatic stellate cells, fibroblasts, biliary epithelial cells, immune cells, and adult stem cells [21], all of which play critical roles in the liver. Proper liver function is highly dependent on the interactions between these varying cell types and hepatocytes; therefore, by incorporating multiple cell types in a 2D culture, it is possible to produce a more physiologically relevant model with tissue characteristics and increased liver functionality [80, 81]. Recently, many co-culture cell systems have been developed. For example, hepatocyte/macrophage co-cultures have been successfully used to model acute responses to septic liver injury and liver regeneration [18, 23, 82]. Culturing hepatocytes with LSECs, in the presence or absence of collagen, allows LSECs to maintain their phenotype and hepatocytes to increase their in vivo-like function also enhanced (e.g., CYP activity) [83]. Similarly, when HepG2 cells are co-cultured with LSECs [84], CYP enzyme induction is enhanced, suggesting that the introduction of endothelial cells influences hepatocyte function in vitro (46). Furthermore, in addition to LSECs enhancing hepatocyte function, similarly hepatocytes support LSEC function [83], influencing LSECs ability to control important repair mechanisms in the liver following injury [85]. Kupffer cell/hepatocyte co-cultures have also been reported, exhibiting responses to a variety of inflammatory stimuli not observed in monocultures [86]. Metabolic functions are also dramatically increased when KCs are cultured with hepatocytes, as demonstrated by Yagi et al. [87]. Various other immune cells have also been co-cultured with hepatocytes [23, 88], such as, for example, the monocytic THP-1 cell line [89]. Huh7 and THP-1 co-cultures expressed pro-inflammatory and stress-related signalling molecules following treatment with troglitazone, a hepatotoxic compound [89]. Furthermore, the co-culture showed increased sensitivity and drug metabolism as compared to Huh7 monocultures [89]. Due to their distinct advantages over hepatic monocultures, co-cultures of liver cells have also been used for the pre-clinical assessment of NBMs. One such study by Esch et al. used a HepG2/C3A co-culture model, incorporated with a Caco-2/HT29-MTX co-culture to form a GI tract-liver system for assessing the uptake, accumulation, and toxicity of polystyrene nanoparticles [90]. Despite the many advantages of co-cultures when compared to traditional hepatic monocultures, these models suffer some disadvantages, summarised in Table 1, and there are fundamental limitations to the number of cell lines that can be cultured together. This issue is associated with others such as determining the appropriate culture environments and conditions, or the lack of essential ECM components, e.g., collagen, which are impacting the cellular phenotype and behaviour and responses to NBMs, as described below.

### Complex liver architecture and how it is reduced in 2D

Another disadvantage of adopting conventional 2D in vitro models is their architecture. Hepatocyte morphology and behaviour are greatly altered due to 2D cell cultures’ inability to reproduce the bio-physical cues of human liver connective tissue [91]. Cell behaviour and phenotype are also dramatically affected by the microenvironment across the cell–cell/cell-extracellular matrix (ECM) interactions. In the human hepatic environment, cells have direct contact with each other and the extracellular matrix due to their intrinsic 3D organisation, which is vitally important for cell signalling [92, 93]. When hepatocytes are cultured in 2D, they lose their 3D interactions and spatial arrangement, and as a consequence their signalling pathways and functions are modified. For the liver to carry out its functions, hepatocytes must exhibit a functional polarised phenotype, with specific transporters localised to both the apical (canalicular) and basolateral (sinusoidal) membrane [23, 24, 28]. In 2D cell cultures, the loss of appropriate hepatocyte polarity is thought to be one of the key factors which leads to inaccurate predictions of toxicity in humans [15]. Notwithstanding, unorganised cell proliferation occurs due to the lack of polarisation induced by the 2D environment, a further cause of inaccurate predictions of toxicity [94, 95].

## In vivo models for liver toxicity screening: advantages and disadvantages

On the other end of pre-clinical assessment sit in vivo studies. In vivo liver toxicity screening tests are vital components of the pre-clinical assessment cascade for NBMs, with the use of animals in pre-clinical research providing not only a basic overview of the fate of NBMs in organs, but also information on dosing and potential systemic toxicities. Currently, in vivo animal studies for NBMs are most often carried out in porcine or rodent models [96], in accordance with the European Commission Directive 2010/63/EU and the EU legislation on the protection of animals for scientific purposes (which protects living non-human vertebrae and foetal mammals from the last third of their normal development).

### Rodent models

As just mentioned, rodent models are one the two most commonly used and preferred mammal species in in vivo studies. Rodent models are useful due to a variety of reasons including their wide availability, low cost, small sizes, and consequent ease of handling; short life span and fast reproduction rate; and abundant genetic resources [97]. The vast majority of NBM formulations have been assessed in rodent models, with studies looking at a variety of implications ranging from liver toxicity to biodistribution to therapeutic effect. For example, a study from Bahamonde et al. assessed the toxicity of 15-nm AuNPs in both mice and rats [98]. Lu et al. used xenograft mice to observe the pharmacokinetics, pharmacodynamics, and toxicity of a PEGylated liposomal formulation of doxorubicin [99], and Recordati et al. have assessed tissue distribution and acute toxicity of AgNPs [100]. Hepatotoxicity and the role of the “gut-liver axis” have been assessed in rats following oral administration of TiO_2_ [101], and the rat model has been used to show that nano-copper induces strong hepatotoxicity, via oxidative stress and inflammation [102]. A large number of studies have assessed the impact AuNPs have on the liver specifically [98, 103–107]. The effects AuNPs have on the liver, among other organs, are also well detailed in a recent review from Kozics et al. [108]. In another study, nude rats have been used to assess the accumulation and biodistribution of transferrin (Tf)-conjugated PEGylated liposomes loaded with doxorubicin [109]. Despite the importance of rodent studies in the pre-clinical assessment of NBMs however, they do have disadvantages associated with them. Despite the genetic similarities to humans, rodent models still show fundamental differences in anatomy, physiology, and immune response [109–112], and they are often criticised for a failure to accurately mimic human disease phenotypes. Comprehensive studies from Seok et al. and de Souza et al. which have assessed the inflammatory responses between humans and mice have shown that genetically changing orthologs in mice demonstrated no correlation to human counterparts [113, 114]. This divergence is further extended to cancer studies, where although humans and mice share a similar risk of developing cancer throughout their lifetimes, approximately 30%, when cancers were characterised there were stark divergences in not only phenotype, but also tumour origin and karyotype [115].

### Porcine models

Following rodent models, pigs, or porcine models, are the second most commonly in vivo model used for NBM testing. Porcine models have many advantages over the smaller rodent models. Many studies have confirmed presence of human-specific cell types in pigs, which cannot be found in rodents [116]. The pig size makes it suitable for both multiple measurement and longitudinal measurement, and there is functional equivalence of various diseases across humans and pigs. Their long life spans make them useful for testing of chronic conditions such as cardiovascular disease, and they also easily adapt to their environmental conditions [117] (cloven-hoof animals also, in general, demonstrate higher sensitivity to pulmonary distress than rodent making these reactions better reproduced in pigs than other models). These factors, coupled with their physical similarities to humans with regard to physiology, anatomy, epigenetics, and immunogenetics, make porcine models ideal for recapitulating liver-specific diseases ailments such as human hepatocellular carcinoma (HCC), and its co-morbidities, including cirrhosis and non-alcoholic steatohepatitis (NASH) [116]. For example, Andrasina et al. utilised pig models to assess the accumulation and effects of liposomal doxorubicin in liver tissues by radiofrequency ablation and irreversible electroporation [118]. Edge et al. have used anaesthetised pigs to assess the pharmacokinetics and biodistribution of novel superparamagnetic iron oxide nanoparticles (SPIONS) [119], and the biodistribution of iron oxide nanoparticles used for drug delivery and imaging in the liver, among other key organs, has also been assessed [120]. The effect on liver morphology induced by various forms of zinc oxide nanoparticles has been assessed in weaned piglets (with some changes detected) [121], and livers have also been harvested from pigs to generate liver slices which were used to study AgNPs [122]. There is also much evidence to suggest that the pig model is very sensitive to detecting NBM-induced complement activation-related pseudo allergy, or CARPA [123, 124]. Whilst not wholly relevant to liver toxicity specifically, some of the most recent and interesting porcine studies are seen with regard to the development of vaccines for COVID-19. Pig models, along with mice, have been used by Kang et al. to study a RBD-mi3 conjugated nanoparticle vaccine candidate. Here, vaccination of both animals with two doses of adjuvanted RBD-mi3 induced a strong nAb response, equivalent in the study to sera from convalescent patients, and 5 to 10 times higher than soluble RBD alone [125]. Despite pig models being a crucial model for assessing NBMs, they are not without their own intrinsic disadvantages. Whilst their size makes them more physiologically similar to humans than smaller animal models, researchers are limited in the specialist facilities needed, i.e., housing, surgery, imaging, and necropsy. There is also greater cost implicated with porcine studies, and also more ethical concerns [117].

### Zebrafish model

In addition to the two models abovementioned, an emerging in vivo model for the pre-clinical assessment of NBMs is the zebrafish, a non-mammal model offering new, practical, and cost-effective opportunities to bridge the gap between in vitro and in vivo studies [126–128]. The recent popularity of this model regarding the study of NBMs is supported by the development of automated, high-throughput readout technologies that can be easily integrated with the zebrafish model [129]. Zebrafish has many characteristics that make it more attractive than conventional in vivo models. Husbandry costs are relatively low compared to rodents, and with the huge financial burdens associated with NBM in vivo studies, this factor is attractive to researchers. Quite possibly the most attractive factor associated with zebrafish is in fact that they can be used without any ethical approval. In accordance with the European Commission Directive 2010/63/EU on the protection of animals for scientific purposes, early stage zebrafish are not protected due to their inability to independently feed [130]. Also, young zebrafish, i.e., zebrafish larvae, are optically transparent, enabling high-resolution imaging of various events in real time [126]. As with all in vivo models, the question of conservation of characteristics and biological features must be asked prior to implementation as a suitable model for NBM assessment. Zebrafish anatomy and physiology are well described in literature [131, 132], with other physiological parameters and organs of relevance to biodistribution and toxicity of NBMs also extensively studied, including the lymphatic system [133], blood components [134], immune [135] and vascular systems [136], and the liver [137]. According to these studies, zebrafish share many essential physiological homologies with humans. Seventy-six percent of human genes have orthologues in zebrafish, thus being relatively comparable to murine or chicken models, which have 84% and 80% respectively [138]. Given the advantages over their rodent counterparts, zebrafish and their larvae are becoming increasingly used as models for the assessment of NBMs, with accumulation, circulation, biodistribution, stability, and toxicity all studied in living zebrafish [126]. Examples of the use of the zebrafish model in the pre-clinical assessment of NBMs include work by Vibe et al., who assessed the toxicity of thioridazine-encapsulated PLGA particles [139], Peng et al., who assessed the release of hydrophobic drugs from cyclodextrin- and dextran-based nanocarriers [140], and Yan et al., who used zebrafish to study photothermal-controlled drug delivery using mesoporous silica nanoparticles [141].

Whilst advantageous due to their aforementioned characteristics, there are still some limitations with this emerging technology in NBM research and answering some specific research questions with regard to NBMs may be problematic in this model. The most obvious issue with zebrafish is the fact that they are not a mammal, making them fall short for disease modelling [142]. Zebrafish are poikilothermic and their developing embryos lack a placenta, meaning that drugs or NBMs may be metabolised in a varied manner or at a different rate when compared to mammals. Gender is also not genetically determined in zebrafish, unless they are altered by hormones [143]. Nevertheless, the choice of the animal model and the awareness of its intrinsic limitations and assumptions are important elements when assessing NBM liver toxicity and accumulation. Despite the deeply rooted assumption among the scientific community that the animal models briefly described above are good predictors of human toxicity, in fact, there is much information which exists to the contrary [144–146]. Only 60% of drugs entering clinical trials are successful in phase I (safety) trials [147], with half of these failures occurring due to unanticipated human toxicity, unseen in animal models.

### The human relevance of in vivo models

Our provocative question is: if animal studies are truly predictive of human toxicities, why are toxicity rated drug attrition rates in human trials so high? Notably, this is not a concern shared only by the authors, and consensus among several scientists is that in vivo animal models are not as accurate predictors of human responses as we have assumed to date [148, 149]. In 2006, a review of 76 different animal studies found that animal results could be replicated in human randomised trials only in 28 cases (37% of the studies), whilst animal results were completely contradicted in humans for 14 cases (the remaining 34 cases remained untested in humans) [150]. A further review of 221 animal studies showed that only in 50% of the case results obtained from animal experiments agreed with those generated by human trials. Here, discordance between animals and humans was found to be caused by both experimental bias and the failure of the animal model to adequately mimic human disease state [151]. When concerned with the ability of animal models to accurately predict hepatotoxicity, the issues are similar. A review of 230 animal studies undertaken in 2000 showed that hepatotoxicity had an extremely poor animal-to-human correlation [152]. This review was not a unique case, and the poor prediction of human liver toxicity by animal models has been reported also by other studies [153, 154]. Several possible reasons have been proposed as to why such discrepancies are seen between animals and humans. The primary reason is linked to intrinsic interspecies differences with regard to both liver physiology and metabolic capacity of animals. Other factors include the heterogeneity of patients’ populations, lifestyle, environmental factors, susceptibility, and pharmacogenetic factors that may make patients more sensitive to adverse reactions than their animal experimental counterparts [155]. Notably, the poor predictive value of animal models can, and has in the past, have catastrophic outcomes for patients, either during clinical trials or months later after a drug was released on the market. One example is the case of fialuridine (1-(2-deoxy-2-fluoro-1-d-arabinofuranosyl)-5-iodouracil or FIAU), a nucleoside analogue that was developed as a potential therapy for viral hepatitis B [156]. Despite not showing any hepatotoxic indications during pre-clinical animal studies undertaken in several animal species (rat, mouse, dog, and cynomolgus monkey) [157], 7 of the 15 volunteers enrolled in the phase I clinical study suffered lactic acidosis and acute liver failure a few weeks into the trial, at doses 100 times lower than those used in the pre-clinical animal studies. Five of these patients died, and two more only survived after receiving liver transplants [156, 158]. Over 20 years later, a retrospective study by the US National Academy of Sciences analysed all pre-clinical toxicity studies of fialuridine and confirmed that in all animal studies undertaken there was no indication that the drug would cause human liver failure. They did however demonstrate that a specific mice model with humanised livers could in fact recapitulate the drug toxicity, again illustrating the importance of being aware of the limitations and assumptions intrinsic within the use of a model [159]. Other examples include Serazone, a serotonin antagonist and reuptake inhibitor (SARI) drug used to treat depression, and Rezulin, a drug used to help control blood sugar levels in type I diabetic patients. Both drugs were recalled from the market for inducing severe hepatotoxicity, which went unnoticed during the pre-clinical assessment as a results of the lack of predictability of the animal models used in the studies [160]. Until recently, Abraxane^®^, an NBM-formulation of albumin-bound paclitaxel, currently used in the clinic to treat metastatic breast cancer and NSCLC, did not come with any specific guideline for its use in patients with liver dysfunction. This oversight led to one patient suffering grade IV febrile neutropenia, grade III mucositis, and grade III nausea/vomiting. As Abraxane^®^ is hepatically metabolised via CYP450 enzymes, specifically CYP2C8, a decreased clearance was observed here due to the patients’ hepatic dysfunction, yielding severe drug toxicity [161, 162]. These severe side effects further demonstrate the need for bio-comparable pre-clinical assessment models that predict adverse outcomes in patients. False positives are another issue in using animal models for liver toxicity screening. False positives can bring to the dismissal of a potentially useful NBM candidate due to liver toxicity in animals, toxicity that is not observed in humans [146]. If false positives cannot be identified early on, they can cause huge loss of both time and money, and overall yield a negative impact on patients’ prognosis and quality of life.

To summarise, it is fair to say that animals do not accurately predict human liver metabolism [148, 154], and although liver toxicity screening in animals is necessary to meet specific regulatory requirements [163], it is now evident that ADME studies on NBMs are in need for complementary experiments using more human-relevant models [164, 165].

## Advanced 3D cell culture models vs conventional pre-clinical methods: advantages and disadvantages

**Fig. 3:**
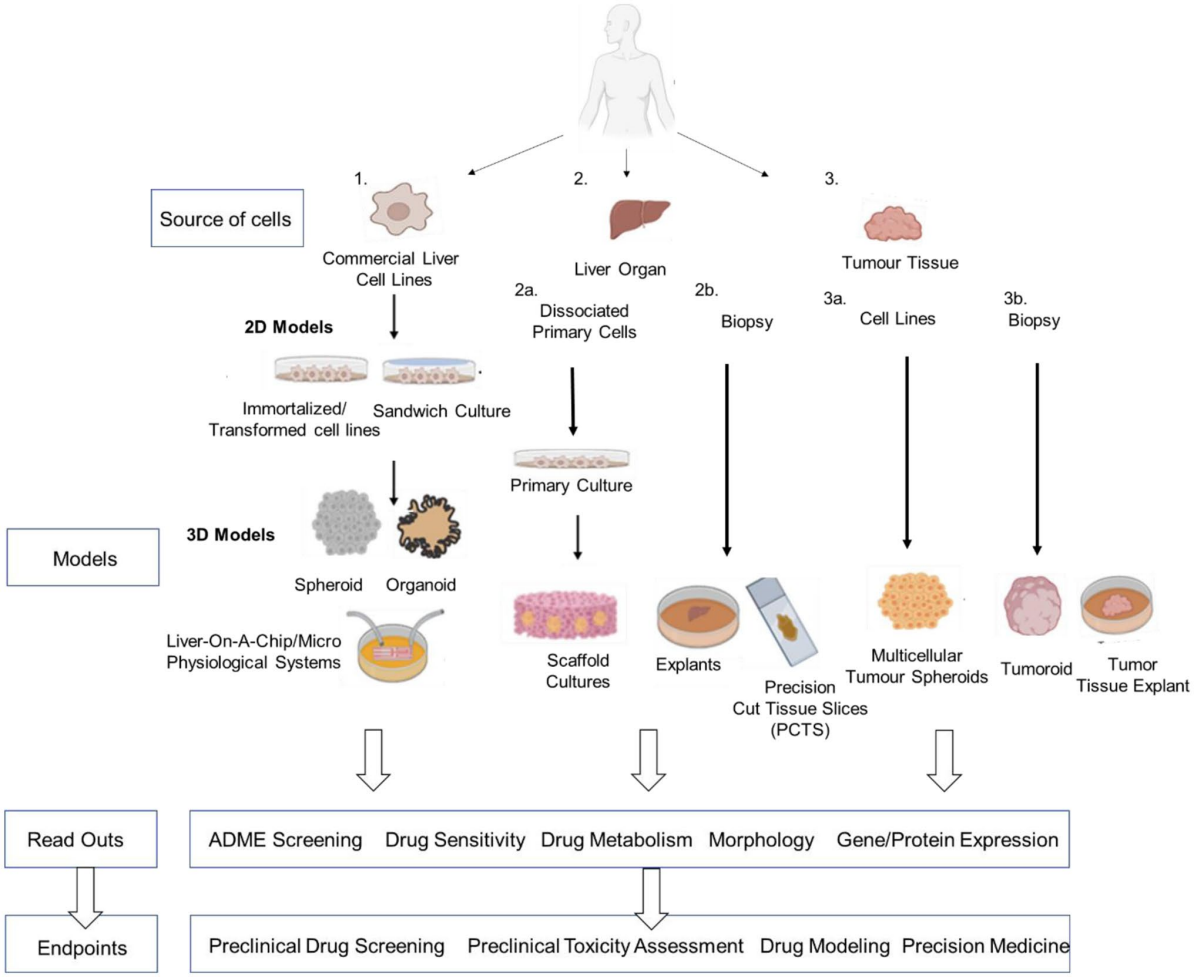
Overview of the various 2D and 3D in vitro models of the liver. Flow diagram illustrates the source of liver cell/tissue, their potential 2D and 3D models which can be formed from both sources, the readouts that can be potentially measured, and their associated experimental endpoints. Figure adapted from Hepatocellular carcinoma, Chapter 3: In vitro models of the liver: disease modelling, drug discovery and clinical applications [14]

In recent years, the scientific conversation has switched towards the concept of adopting more human-relevant pre-clinical research by focusing on reliability, accuracy, and relevance of the testing methods adopted. It is now suggested that animal studies could be either replaced (“1R principle”) or at least widely reduced (as part of the “3Rs principle” of replacing, reducing, and refining the use of animals in scientific research) with other more reliable methodologies. Such methodologies, generally referred to as new approach methodologies (NAMs), may act as huge time- and cost-saving technologies in the NBM development pipeline, provided they are validated and their efficacy in pre-clinical testing is proven. For NBMs, as previously reported by some of the authors [166–172], 3D in vitro cell-based methodologies are now seen as a vitally important emerging technology in the NBM pre-clinical assessment cascade [11]. In recent years, sophisticated, physiologically relevant 3D liver models have been developed to predict more accurately the hepatotoxicity of NBMs [173]. These include models formed not only from immortalised cell types (i.e., spheroids, organoids and liver-on-chip), but also whole organ (i.e., scaffold cultures, precision-cut tissue slices, and explants) and tumour tissue sources (i.e., tumouroids, multicellular tumour spheroids, and tumour tissue explants), as illustrated in Fig. 3, which also compares both models with respect to cell/tissue source, readouts, and endpoints. These models not only recapitulate whole organ physiology compared to 2D models, but they are also suitable for repeated exposures and chronic drug testing, an important consideration for NBM pre-clinical assessment [19]. 3D hepatic models have been shown to exhibit similar patterns of NBM transport, adsorption, and distribution that are observed in the human liver [174, 175], and they also remain both viable and functional for lengthy culture periods, a characteristic which renders them useful for repeated-dose and chronic hepatotoxicity assessment of NMBs [176–178]. Outside of their uses in toxicity screening of NBMs, these newer models also give better insights into the pathogenesis of liver diseases such as NAFLD and steatosis, therefore unlocking the potential for shifting the in vitro NMB testing from hepatotoxicity testing to simultaneous toxicity and efficacy screening [179]. Key differences between 2 and 3D models, with respect to morphology, in vivo-likeness, and response to materials, among other parameters are described in Table 2. Table 3 details associated advantages and disadvantages of the various hepatic models. In the following sections, some of the most common 3D in vitro models for hepatotoxicity screening are described in detail.

**Table 2:** Key differences between 2D and 3D culture, regarding morphology, response to materials, in vivo likeness, and other key parameters

**Property**	**2D liver cell model**	**3D liver cell model**
**Morphology and architecture**	Sheet-like, flat, stretched cells grown in monolayers; do not mimic natural architecture of liver	In vivo-like cell shape; high similarities to in vivo liver architecture
**Cell proliferation**	Cells proliferate at a higher rate than in vivo	Cells proliferate at faster or slower rate than 2D culture, depending on cell type/3D system
**Protein/gene expression**	Often display different expression levels to human liver tissues	Protein/gene expression levels similar to those found in human liver tissues
**Access to oxygen, metabolites, nutrients, and signalling molecules**	Unlimited access	Access is defined by the 3D morphology of the cultures as per in vivo conditions
**Cell–cell interactions**	Cannot recapitulate cell–cell and/or cell-ECM interactions due to flat morphology	Appropriate interactions between cell–cell and cell-ECM are established
**Multicellular composition**	Co-cultures can be formed; number of cell types co-cultured is limited	Tissue composition can be fully replicated (e.g., organoids)
**Sensitivity to stimuli/hepatotoxins**	Sensitivity is often not comparable to the in vivo liver tissue	Better predictors of in vivo responses
**Exposure to NBMs**	All cells are equally exposed to NBMs	Depending on culture morphology, NBMs may not penetrate the core and reach all cells, as per in vivo conditions
**Reproducibility**	Reproducible high-performance and simple but highly reductionist	Reproducibility depends on method, can be user-dependent, but it can be optimised
**Cost of maintaining culture**	Cheap, all reagents/materials commercially available	Often more expensive, time consuming, increased batch-to-batch variation

**Table 3:** Advantages and disadvantages of commonly used 3D culture methodologies

	**Advantages**	**Disadvantages**
**Spheroids/multicellular tumour spheroids (MCTS)**	Easy to culture Mimic in vivo-like cell–cell and cell-ECM interactions Scalable and high-throughput (HTP) compliant Easily extracted for further experimentation	Size variability Limited diffusion if large Necrotic core formation Agglomeration Take time to form and show functionality
**Scaffolds/hydrogels**	Hydrogels: in vivo-like 3D interactions Used to study cell aggressiveness/metastatic potential Scaffolds: can be combined with functional tests	Hydrogels: size/shape variation. Hard to reproduce Difficult cell extraction Scaffolds: cells can flatten/adhere to scaffold Difficult materials can affect growth
**Organoid/Tumouroid**	In vivo-like architecture In vivo-like complexity Patient specific Replicate in vivo-like cell interactions	Complex to culture Variation Less amenable to HTS Needs much optimization/validation May lack certain cell types/vasculature
**Liver-on-chip/microphysiological systems (MPS)**	In vivo-like architecture In vivo-like chemical/physical gradients, microenvironment	Flow of medium may disrupt cells Difficult to adapt to HTS Lack vasculature
**Explants/tumour explants**	In vivo-like architecture In vivo-like complexity Useful in modelling disease	Variation between donors Difficult to obtain Complex to culture/expensive to maintain Lack long-term viability

### Sandwich cultures

The earliest attempts to recapitulate the complexities of the in vivo liver environment date back to the work of Dunn et al., in 1989, when a hepatocyte sandwich culture model (SCH) was developed using adult rat hepatocytes and a single layer of collagen [180–182]. Nowadays, SCH liver models commonly incorporate two layers of a component of the liver ECM (e.g., collagen) or a naturally occurring ECM like Matrigel™ [183, 184]. Whilst still reductionist due to the lack of the cell–cell interactions that can be observed in the in vivo liver organ, SCH models do allow the establishment of cell-ECM interactions, an important step up from conventional 2D monocultures. Because of this, a more in vivo-like hepatic environment is promoted, with maintenance of cellular polarity and tissue-like metabolism, formation of gap junctions, and normal levels of key liver-specific proteins and substances (e.g., albumin, urea, bile acids) observed [95]. In a SCH, formation of intact and functional canalicular networks also occurs and can be maintained over several days in culture [48, 185]. All of these characteristics make SCH models relatively good in predicting human hepatotoxicity, making them useful tools for determining transport, interactions, and hepatotoxicity of NBMs [48].

### Scaffold-based cultures

A popular method employed for 3D culture of hepatocytes is the use of culture matrices as scaffolds for cell growth [23]. Matrigel™ has proven to be very useful in the culture of hepatocyte cell lines for modelling the in vivo liver environment, with a study undertaken by Molina-Jimenez et al. demonstrating that culturing Huh-7 cells in Matrigel™ as 3D models allows prolonged viability (useful for chronic toxicity studies), hepatocyte polarity, and functional transporters [183]. Matrigel™ has also been used to culture primary human hepatocytes (PHH) in 3D environments, with Bell et al. [186] proving that when 3D and 2D PHH cultures were formed from the same donors, 3D spheroids were more functionally stable and exhibited a greater sensitivity in detecting hepatotoxins as compared to their 2D counterpart.

Collagen is another natural ECM component commonly used as scaffold for growing hepatocytes in 3D. When cultured with collagen, many hepatocyte cultures exhibit improved urea production, albumin synthesis, and CYP450 activity [187]. In recent years, advancements in material chemistry and material fabrication have led to the design of 3D culture materials that accurately represent the chemistry, geometry, and signalling found in the liver ECM. These synthetic substrates have been used for studying liver toxicity of NBMs, with Kotov et al., forming highly viable HepG2 spheroids with intact junctions using hydrogel scaffolds. Interactions between hepatocytes and CdTe NPs and AuNPs were studied using this model, and it was observed that the toxic effects of both materials were significantly reduced in 3D culture when compared to 2D, with phenotypic changes and the tissue-like morphology identified as the major factors implicated in these differences [188].

A further scaffold-based culture technique worth mentioning is 3D bioprinting, which uses manufacturing techniques and computer-assisted technology to form 3D structure from cells and biomaterials, with the overall goal of printing functional tissues and organs [189]. The primary advantage of bioprinting is controlled cell distribution, something which can be an issue when seeding cells onto larger scaffolds. In 2010, the first reported bioprinted liver was formed from HepG2 cells encapsulated in alginate hydrogel and was used for drug metabolism studies [190]. A second notable bioprinted 3D liver model was formed from human-induced pluripotent stem cell (hiPSCs)–derived hepatic progenitor cells, adipose-derived stem cells, and human umbilical vein endothelial cells (HUVECs). This model was maintained in culture for over 20 days, and exhibited enhanced hepatic morphological organisation, liver-specific gene expression, increased albumin and urea production, and enhanced CYP450 induction [191]. More recently, Nguyen et al. established a novel bioprinted human-derived mini-liver formed from HUVECs, hepatic stellate cells (HSCs), and human primary hepatocytes for the in vitro assessment of liver toxicity induced by clinical drugs [192, 193]. This unique tri-culture 3D model not only allowed for a more relevant assessment of cytotoxicity, but also enabled the measurements of responses specific to each of the cell types.

### Cell spheroids and multicellular tumour spheroids (MCTS)

**Fig. 4:**
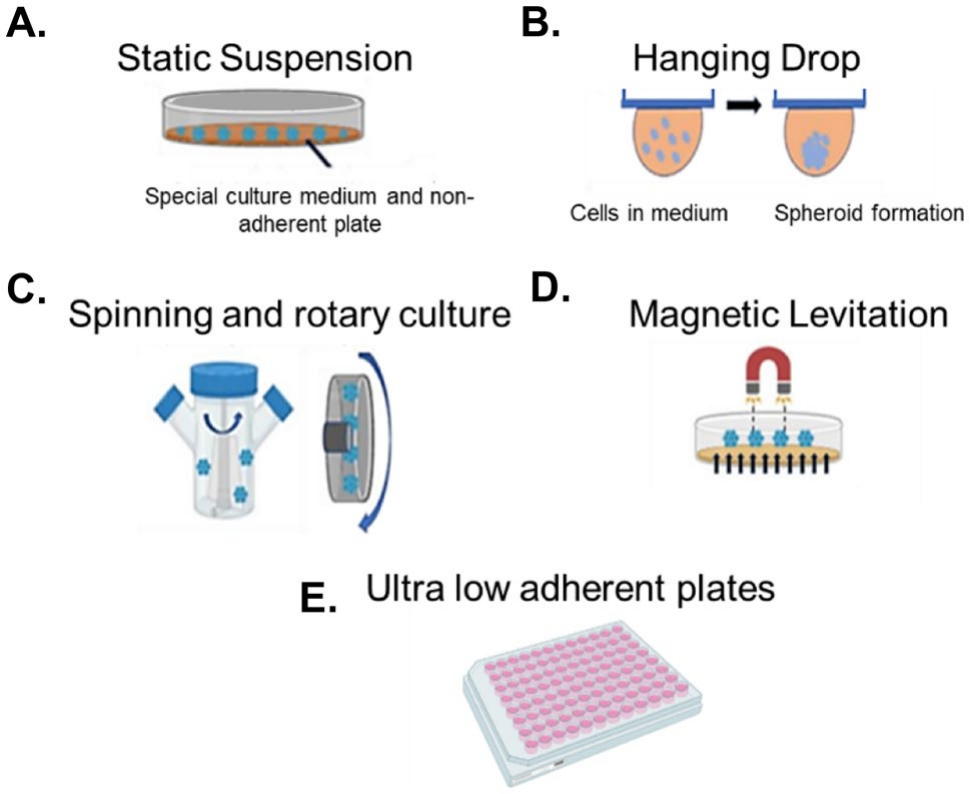
The most common methods for spheroid formation, cultivation, and growth. A variety of different techniques for 3D spheroid production exist, each varying in complexity and ease of use. Common techniques include (**A**) static suspensions, or the liquid overlay technique, with spheroids formed from interruption of cell adhesion on non-adherent surfaces, (**B**) hanging drops, undertaken using commercial systems like InSphero™ Gravity Plus™, or using upturned petri dishes, whereby cells are seeded in small drops in medium and spheroids form due to gravitational forces, (**C**) rotary and spinning cultures, formed in vessels specifically designed to prevent cell adhesion, (**D**) magnet-assisted cultures or magnetic levitation, where cells are magnetised in culture, often by using nanoparticles, and pulled towards a magnet on top of the culture vessel, and (**E**) ultra-low attachment (ULA) surfaces and plates. Each technique is based on the principle to force cells to self-aggregate and compact into spheroidal 3D microtissues

One of the most popular and widely used 3D methodologies for culturing hepatocytes to date is the formation of cellular 3D spheroids [194]. 3D spheroids are small, round clusters of cells which can self-assemble naturally in non-adherent environments, closely mimicking cell–cell interactions, as well as biological processes occurring in an in vivo scenario [195]. In recent decades, they have gained increased attention in both drug discovery and tissue engineering due to the huge array of advantages they have over existing 2D monolayer cultures, and researchers are now looking towards 3D culture and spheroid models increasingly to bridge the gap between reductive 2D monolayer cultures and in vivo animal models. There are a wide variety of methodologies for inducing the formation of cellular spheroids through self-aggregation (presented in Fig. 4), each varying in complexity and ease of use.

Common techniques include ultra-low attachment (ULA) surfaces and plates [196], hanging drop techniques [197], the use of rotary/spinner or rocked culture vessels [198], and the use of micro-patterned culturing surfaces [199]. Each of these methods is advantageous for producing hepatic spheroids as they allow hepatic cells to self-aggregate and form microtissues as they would in an in vivo situation, without the need to incorporate into scaffolds. Cells form direct cell–cell contacts, produce their own ECM, and maintain viability longer, overcoming some of the most commonly seen negative effects of conventional 2D and scaffold-based 3D models [200]. For example, HepG2 spheroids have been shown to exhibit a strong in vivo-like cellular organisation, enhanced production of albumin and urea, and an upregulation of many genes which play essential roles in lipid metabolism and xenobiotics [201]. Spheroids formed from C3A cells show increased liver-like functionality and are more sensitive to hepatotoxins when compared to C3A 2D cultures [202]. PHH can also be maintained long term in 3D spheroids, with these spheroids remaining viable and functional for over five weeks [203]. This helps to overcome one of the primary limitations of PHH cells, i.e., long-term viability. Sustained phase I and II enzyme expression is also observed in 3D PHH spheroids, along with increased albumin and urea secretion, and expression of various liver-specific markers [204, 205]. Formation of bile canalicular-like networks is also observed, demonstrating hepatocyte polarisation [204, 205]. 3D liver spheroids can be grown from hepatocytes on their own (as in the examples above) or from hepatocytes in co-culture with other liver cell types, i.e., multicellular liver spheroids. Multicellular spheroids, formed from immortalised hepatic cell lines or PHH and other non-parenchymal liver cells, including liver sinusoidal endothelial cells (LSECs), Kupffer cells (KCs), or hepatic stellate cells (HSCs), provide an in vitro model with a structure and function which closely mimics the human liver [206]. For example, hepatocyte-HSC co-culture spheroids exhibit the same liver-specific morphological and structural properties as it is observed in monocultures. Additionally, they exhibit a 30% increase in albumin secretion, maintained up to two months in culture, and enhanced CYP450 enzyme expression and activity [195, 207]. Co-culture models of KCs and LSECs remain viable for up to 35 days and exhibit enhanced expression of transporters and sensitivity to hepatotoxins [204], making them useful models for liver toxicity screening.

One useful application of hepatic spheroids is in fact in the pre-clinical assessment of NBMs. They can provide an understanding of the NBM toxicity in the context of absorption and penetration within the liver tissue. Also, hepatic spheroids have a longer life span, rendering them useful for repeated dose or chronic toxicity testing, a major advantage over traditional in vivo studies [196, 208]. Hepatic spheroids have proved useful in the pre-clinical assessment of many NBMs [188, 209, 210], and are often used to study conditions such as drug-induced liver injury (DILI) [203], which cannot be accurately predicted using animal models. Hepatic multicellular tumour spheroids (MCTS), i.e., 3D spheroids formed from hepatocarcinoma cell lines, have successfully been used for assessing NBM cytotoxicity and for predicting in vivo anti-tumour activity, as detailed by Mikhail and co-workers. Here, a MCTS model was treated with docetaxel (DTX)-loaded micelles, showing that spheroids were significantly more resistant to treatment in comparison to the corresponding 2D monocultures and responded in an in vivo-like manner, demonstrating that MCTS are viable and in vivo-like platforms for evaluating the impact of NBMs, in conditions which closely mimic in vivo tumour microenvironment [211].

### Liver organoids

In recent years, liver organoids have emerged as a highly relevant and useful alternative in vitro model with excellent potential for disease modelling and drug and NBM screening. These models are advantageous over conventional in vitro models due to their long-term genetic stability, in vivo like organisation, and their ability to maintain the cellular crosstalk and behaviour of their primary cell counterparts [212, 213]. Formed from induced pluripotent stem cells (iPSCs), embryonic stems cells, hepatoblasts, or organ-specific adult tissue-derived cells (both healthy and diseased), which have the ability to self-assemble and differentiate, these functional 3D hepatic models serve as useful platforms to address a wide variety of research questions, ranging from hepatic development and regeneration, to metabolism and detoxification, and are also extremely representative model diseases of the liver including NASH and NAFLD [213], acting as an excellent resource for studying the human liver in ways that were previously very difficult. Up until recently, 2D hepatocyte cultures were the first in line with regard to drug and NBM metabolism and toxicity screening. However, due to impaired CYP450 activity, they are unstable and lack functionality. As detailed by Mun et al., hepatic organoids help overcome these limitations as they have been shown to express phase I drug metabolism and phase II detoxification enzymes. In this study, following treatment with omeprazole, CYP1A2 and CYP3A4 induction was observed in hepatic organoids. Additionally, when treated with the hepatotoxic drugs troglitazone and APAP, in conjunction with 2D hepatocyte cultures, the organoids exhibited a markedly higher sensitivity than the 2D cultures [214]. These organoids have been extensively investigated as most recently reported in the comprehensive reviews published by Prior and co-workers [213, 214].

Liver organoid models have been applied to the pre-clinical assessment of the biocompatibility and toxicity of NBMs. For example, Kermanizadeh et al. have assessed the hepatotoxicity of AgNPs using hepatic organoids formed from human primary hepatocytes and NPCs. Here, following organoid characterisation, various parameters, including cytotoxicity and genotoxicity, were assessed following NBM treatment [53]. Hepatic organoids have also been applied to the assessment of complex NBM systems, such as the liposomal DNA origami nanosystem (LSTDO) designed by Palazzolo et al. Here, doxorubicin was loaded inside the LSTDO, and the system was tested in both murine hepatic organoids and in vivo xenograft mice to determine if such a system would be clinically relevant and to determine if it improves on the current cancer treatment. Before beginning the in vivo mouse study, biocompatibility was ensured by undertaking toxicity screening using the mouse-derived hepatic organoid model, and it was found that the DNA origami internalization into liposomes negated many of the toxic effects of free DNA origami. Furthermore, it was found that the combined properties of the liposomes and DNA origami facilitated the introduction of doxorubicin in a biocompatible manner, improving drug accumulation at the tumour site, and inhibiting tumour growth in the mice [215].

To the best of our knowledge, the wider use of human-derived liver organoids in the pre-clinical assessment of NBMs is still in its infancy, and little information is currently available on their wider applications for screening next-generation NBMs such as liposomes or SPIONs. Despite the huge interest in liver organoids, they are not exempted from shortfall and limitations, such as the accurate recapitulation of the in vivo ECM. Others are the lack of a native microenvironment, a problem which can hinder the study of stem cell and niche interactions, the lack of necessary growth factors, and an inability to accurately model the immune response. One possible solution for overcoming some of these issues could be the application of microfluidics to these models, as we are discussing in the following sections.

### Ex vivo models of the liver: precision-cut tissue slices, whole organ explants, and tumour explants

Ex vivo models include precision-cut tissue slices (PCTS), whole organ explants, and tumour tissue explants. PCTS are generated by cutting slices of a defined thickness from viable liver tissue, often from rodent tissues but in more recent years also from humans [216], with human tissues for PCTS normally obtained from either explanted tissue, partial hepatectomies, tissue unsuitable for transplant, or offcuts from surgery (i.e., diseased liver obtained from cirrhotic or fibrotic patients) [217]. PCTS allow retaining tissue structure, morphology, and intracellular polarization for up to five days in culture (15 with optimal culture conditions) [218]. This offers a model characterised by strong in vivo relevance and with great relevance for hepatotoxicity screening. In PCTS, the multicellular histoarchitecture of the liver environment is maintained, including liver-infiltrating immune cells [219]. PCTS are also useful due to their reproducibility, ease of maintenance, and low cost, making them valuable tools for assessing many elements of various liver diseases [217]. A number of studies have demonstrated that interactions between specific liver cell subtypes are conserved in PCTS, with Olinga et al. illustrating the interactions between Kupffer cells and hepatocytes [220]. Moreover, the ECM, which is notably absent in hepatocyte 2D cultures and 3D spheroids, is also conserved, ensuring the regulation of important cellular functions by associated hormones, cytokines, and growth factors [221, 222].

To date, a number of studies have used PCTS for NBM assessment, as PCTS make it possible to test NBM in a multicellular context in a single in vitro model, which closely resembles in vivo conditions. Dragoni et al. investigated the uptake and toxicity of AuNPs using rat liver PCTS. Here, uptake of AuNPs was observed in hepatocytes, Kupffer cells, and endothelial cells in the liver slices. A more recent publication from Bartucci et al. used, for the first time, human liver slices to investigate ex vivo the NBM behaviour in the liver and to investigate the basic mechanisms of NBM interactions in real time. The findings determined that, as per in vivo conditions, Kupffer cells accumulated large amounts of NBMs, which interestingly move within the tissue slices to the borders [223]. However, despite the usefulness of PCTS in the pre-screening of NBMs, it is worth noting that they cannot be used in a high-throughput manner and rapidly loose functionality days into culture, factors which somewhat limits their usefulness and application [224].

A further advanced ex vivo model, whole organ explants, which can either be suspended in culture medium or embedded in ECM substrates, offers in vivo-like 3D architecture and gene expression which can be maintained up to five days in culture [225]. Whilst conventional studies of the mechanisms and progression of liver disease normally focus on a selection of liver cells and cell culture techniques, whole organ explants offer great advantages over these methods as they not only contain all liver cell populations, but also have their 3D architecture intact. In the past, whole organ explants have been used for studying alcohol-induced liver injury, by exposing them to ethanol [226]. In a similar manner, tumour liver explants can also be used as 3D ex vivo models of HCC for drug efficacy screening [227, 228]. A further study by Piera et al. used liver explants from mice inoculated with a HCC cell line, Hepa1/A1, to study the antineoplastic potential of a well-known antioxidant, Citozym [229]. Despite their many uses and in vivo relevance, whole organ and tumour liver explants do have many limitations associated with them. These include the lack of reproducibility due to inter-donor heterogeneity, lack of viability long term, and issues with availability, rendering them unfeasible models for HTP assays or any chronic toxicity screening [230]. Whilst they are becoming more widely used for modelling disease and for assessing material toxicity, information regarding their applicability to NBM pre-clinical assessment is still scarce.

### Liver-on-chip and microphysiological systems (MPS)

Liver-on-chip 3D can be described as an in vitro hepatic microphysiological system which aims to recreate the physiological conditions of liver tissue on a microscopic scale [231]. Liver-on-chip models are ideal as higher-throughput systems that are capable of mimicking conditions of hepatocytes and the dynamic physicochemical hepatic environment. One example of liver-on-chip for in vitro screening of hepatotoxins is the 3D HepaTox Chip, designed by Toh et al. in 2009 [232]. It is considered as one of the first demonstrations of a microfluidic system that could accurately predict hepatotoxicity, with data obtained using this model strongly correlating to in vivo data [232]. Here, hepatocytes maintained a comparable, if not higher, concentration of phase I and II metabolic functions when compared to monocultures. After 24 h in culture, basal CYP1A1 and CYP1A2 levels were approx. threefold higher in the HepaTox Chip than in multi-well plates [232]. Other research undertaken using C3A cells and HepaTox Chip showed that multiple liver-specific functions including albumin synthesis, gluconeogenesis, and ureagenesis were at almost comparable levels to those of primary hepatocytes [233]. Some of the most recent 3D culture advances have been in this area, and many integrated liver-on-chip microsystems have been developed which reproduce many key structural, functional, biochemical, and mechanical features of living organs, in a single, small device [179]. Liver-on-chip systems bear a close resemblance to the liver sinusoid, with endothelium separating hepatocytes and a constant flow of nutrients, metabolites, and oxygen through the microfluidic channel. Microfluidic liver-on-chip models can overcome the issue of a lack of flow seen with static cultures, allowing for a continuous perfusion of culture medium, nutrients, and/or test compounds to the cell monolayer or cell spheroids, generally improving not only viability and life span of the cells in question, but also their metabolic performance [234, 235].

Other more sophisticated systems also exist, including hollow-fibre bioreactors. Here, cells are seeded on complex scaffolds with a consistent flow of culture medium present. One such study from Gerlach et al. successfully cultured human primary hepatocytes and non-parenchymal cells (NPCs) using this method, with viability maintained for at least three weeks, with vascular cavities and bile duct-like, canalicular structures also visible [236]. Over the years, several microfluidic systems have been developed, with many of them now commercially available through companies like Mimetas, TissUse, and Emulate, among others, therefore facilitating their use as emerging hepatic technologies. These novel microfluidic hepatic models have also acted as a new platform for testing NBMs [90, 237, 238]. As NBMs exhibit differential behaviour depending on whether they are under static or flow conditions, the development of dynamic microfluidic systems is vitally important for accurate determination of NBM toxicity [239]. A primary disadvantage of conventional in vitro models is the issue of providing a constant flow of culture medium/nutrients to the cells, and the artificial way medium is renewed sequentially, to either keep cells alive and healthy, or to introduce NBMs for toxicity screening. The addition of NBMs and culture medium results in non-steady state conditions because of diminishing substrate concentrations, accumulation of product, and other issues such as evaporation over time [240]. Notable examples of this include a study from Li et al., who developed a 3D microfluidic hepatocyte chip for assessing the hepatotoxicity of superparamagnetic iron oxide nanoparticles (SPIONs). Here, primary rat hepatocytes were used to fabricate a three-layer chip which tested SPIONs in short- and long-term culture [241]. A further study from Liu et al. incorporated electrospun fibres in a PDMS microfluidic chip and used this device to culture primary hepatocytes for assessing AgNP hepatotoxicity. Here, hepatocyte behaviour was studied, and it was determined that by using this platform under optimised flow conditions, specific hepatocyte functions including polarity and biliary excretion were restored and maintained for up to 15 days. Hepatocytes under a 10 μL/min flow rate also produced sensitive and consistent toxicity responses to AgNP, demonstrating the applicability of this model for the in vitro toxicity screening of NBMs [242].

It is also possible for liver-on-chip models to be incorporated into a microphysiological system (MPS), where several organs-on-chips are interconnected [243]. Initially, MPS have been developed as a mean for increasing efficiency, speed, and safety in the pre-clinical development and assessment of pharmaceuticals. This was born from the inability of 2D monocultures of immortalised cell lines or in vivo animal studies to sufficiently recapitulate the dynamic of drug-drug, drug-organ and drug-organ-organ interaction in humans. More recently, MPS have been utilised to assess efficacy and safety of NBMs in one single, compact in vitro platform, ultimately advancing the translation of these materials [244]. A recent example of MPS where a GI/liver MPS incorporating co-cultures of Caco-2/HT29-MTX and HepG2/C3A liver cells has been successfully applied for investigating first pass metabolism of high doses of polystyrene nanoparticles intended for daily human consumption [90]. This study determined that despite the nanoparticle permeability across the GI barrier was low, the single nanoparticles and small clusters which did in fact pass through the GI barrier induced aspartate aminotransferase (AST) release in the liver cells, indicating potential liver injury. Overall, sophisticated MPS are a unique model that can offer fundamental understanding of NBM effects in the human body [245].

Despite the wide array of benefits associated with microfluidic models, due to their complex nature they are not without fault. Disaggregation and loss of cells can occur due to the constant perfusion of the culture medium, especially as cells proliferate and reach confluence. Additionally, the effect that sheer stress has on the cultured cells is not fully understood yet [246]. Finally, the large set-up and maintenance costs associated with these models and systems, as well as the large cell densities needed to build the models and the associated issues with their handling and analysis, are impacting or limiting the uptake of such complex platform technologies.

## Advanced 3D cell culture models in pre-clinical testing: translational approaches

This review thus far has presented the many challenges associated with the screening of NBM-induced hepatotoxicity, in depth, ranging from the advantages and disadvantages associated with conventional in vitro and in vivo models, to the new, emerging 3D technologies in this field. The latter offer many exciting opportunities for evaluating the accumulation and hepatotoxicity of NMBs in a human-relevant manner. Not only do they have the potential to closely mimic the specific phenotype and organotypic function of the human liver tissue, but also allow a more sensitive and physiologically relevant assessment of the fate and toxicity of NBMs [188]. They also yield more cost- and time-effective NBM studies and have greatly benefited the wider scientific community since their conceptualisation and development. The effects triggered by NBMs in 3D models differ greatly for those observed in conventional 2D monolayer cultures, a phenomenon demonstrated in many studies, including one by Eljie et al., who used HepG2 cell spheroids for assessing the genotoxic and cytotoxic potential of three nanoparticles, namely silver, zinc oxide, and titanium oxide [209]. 3D cultures are indeed a much more faithful representation of the in vivo hepatic environment when compared to conventional monocultures. Furthermore, these models represent an attractive opportunity in keeping with the 3Rs concept, as a more human-relevant platform or as replacements to animal studies. This is particularly relevant in the EU, as the European Medicines Agency (EMA) has supported the implementation of both Directive 2010/63/EU [130], legislation on the protection of animals used for scientific purpose, as well as the 3Rs principle of replacement, reduction, and refinement, which they have supported from as early as 1986 (Directive 86/609/EEC) [247], when the first legislation for the protection of animals used in scientific research was implemented. Since then, the EMA has not only promoted the ethical use of animals in pre-clinical testing, but now also encourages non-animal alternatives and strives to improve the welfare of animals when their use can’t be avoided. Directive 2010/63/EU aims to harmonise all animal research legislation through all EU member states, ensuring that high standards of both scientific research and animal welfare are met. In the EU to date, animals can only be used in research where convincing justification has been put forward, whereby the research benefits outweigh an animal suffering, and when the specific objectives of a study cannot be met using non-animal alternatives. At all times, these strict ethical guidelines must be met. To date, a variety of advanced liver technologies have been successfully utilised in many areas, and in many parts of the world. Some specific examples of these are detailed below.

### Complex liver models in the USA

Organovo, a San Diego–based company, has developed a bioprinting process whereby liver cells are extracted from donor organs and turned into printable bio-ink [193, 248] to build up small sections of liver tissue. Today, this technology is expected to revolutionise the drug safety screening. In December 2017, for example, the FDA granted orphan drug status to Organova for their treatment of alpha-1 antitrypsin (A1AT) deficiency based on tests carried out using their 3D bioprinted liver tissues. It is safe to say that the revolution of bioengineering complex liver models is well and truly underway, and in recent years we have witnessed superior manufacturing and machine development, improved bio-inks, and a huge influx of published literature which leverages 3D bioprinting as one of the primary tools which will revolutionise and strengthen regenerative medicine in the coming years. In the USA and founded in 2009, the Wyss Institute for Biologically Inspired Engineering at Harvard University is at the forefront of liver-on-chip research. Formed from the understanding that research “can’t change the work unless it never leaves the lab” [249], the Wyss Institute is a cross-disciplinary biomedical engineering research institute focusing on the development of bioprinted material and devices which can be utilised for applications in manufacturing, energy, robotics, architecture, and the healthcare sector. The Wyss Institute is also at the forefront of organ-on-chip technology. In the last decade, the Wyss Institute has developed and validated more than ten different organ-on-chip models, including the liver, and has also engineered a device which automates chip processes and can link the different organ-on-chip models fluidically to form “body-on-chip” models which recapitulate whole body physiology whilst allowing high-resolution analysis and imaging. The Wyss Institute has successfully translated these innovations into commercial technologies, and has leveraged financial support from the FDA, National Institutes of Health (NIH), and the Defense Advanced Research Projects Agency (DARPA). These technologies have also been refined and validated for the clinical and market need, and are now used for testing existing medicines and modelling various diseases, in partnership/conjunction with various major pharmaceutical companies [249].

### Liver models from a European perspective

The move towards more complex liver models has also been reflected in Europe, in one of the latest EU Horizon 2020–funded projects, ORGANTRANS (controlled ORGANoids transplantation as enabler for regenerative medicine TRANSlation). This project aims to develop a 3D printed liver tissue platform to potentially replace liver transplantation for patients with end-stage liver failure, using biofabrication knowledge from the bioengineering company regenHU. Coordinated by CSEM, the Swiss research and development centre, the ORGANTRANS project consortium, made up of two transplantation centres and eight partners, generates novel techniques which can not only be applied to hepatic systems but also other organ systems in regenerative medicine and will cover the complete cycle of development from cell sourcing and tissue bioengineering to clinical trials. Another Horizon 2020–funded project, PATROLS (Physiologically Anchored Tools for Realistic nanomaterial hazard aSsessment), also aims to develop more physiologically relevant in vitro 3D GIT, lung, and liver models which may be incorporated into the pre-clinical testing of nanomaterials. This project was launched in January 2018 and will run for 3.5 years and involve 24 partners across not only the EU, but also the USA and Asia. To date, the PATROLS project has delivered outcomes which include the application of 3D HepG2 spheroids to the genotoxicity screening of NBMs [250, 251], the development of complex co-culture hepatic 3D models for NP toxicity screening [252], various in vitro lung systems for assessing nanomaterial-induced lung toxicity and inflammation [253–255], and a GIT model [256]. Complex liver technologies, including functional, geometric liver models, have also been utilised in Europe by the Max Planck Institute for studying the varying stages of liver disease [257].

### Outside the USA and EU: South America, China, and Japan

With regard to outside the EU and the USA, in the last year, scientists at the Human Genome and Stem Cell Centre (HUG-CELL) at the University of Sao Paulo (USP), funded by the Sao Paulo Research Foundation, have succeeded in forming hepatic organoids from induced pluripotent stem cell (iPSC) bio-ink, which are not only capable of performing liver-specific functions such as bile secretion and storage of vitamins and proteins, but also have prolonged tissue survival over a long culture period in vitro. Using their method, hepatic tissue can be created in the lab in 90 days, and it is expected that this technology will, in the coming decades, become a feasible alternative to organ transplantation [258]. In China, a collaboration between the Department of Liver Surgery at Peking Union Medical College (PUMC) Hospital, the Chinese Academy of Medical Sciences, Beijing, and the Department of Mechanical Engineering, Beijing, has led to the development of 3D bioprinted hepato-organoids (3DP-HOs), constructed from HepaRG cells and bio-ink, using 3D printing procedures in order to overcome the shortage of organ donors, a critical issue for patients with end-stage organ failure. Following implantation into mice models of liver injury, the hepato-organoids in this study matured to display increased synthesis of liver-specific proteins and drug-metabolism activity. The formation of functional vascular systems was also observed, enhancing liver function and material transport further. Perhaps the most important finding was the fact that the 3DP-HOs improved survival of the liver injury mouse model. This study has further emphasised the potential value of 3D bioprinting in the regenerative and transplant medicine field, with this technology allowing the implantation of artificial organs into the human body, ones that demonstrate normal physiological function in vivo, and ones which may offer novel solutions to treat disease [259]. Also, in China, scientists at the Hanghou University of Science and Technology have developed a technology called Regenovo, a medical grade 3D bioprinter which to date has been used to produce numerous gelatinous and semi-transparent 3D bioprinted kidneys, ear cartilage, and micro livers using scaffolds containing live cells, inorganic materials and hydrogels, and medical grade polymers. This printer uses an 80-µM printer nozzle, which allows the printing of cells up to five times the number of normal cells, with cells remaining viable for up to four months. Cell damage rates are also extremely low, with approximately 90% of cells surviving the printing process [260].

Similarly, in Japan, researchers from the University of Tokyo have also successfully bioprinted scaffold-free 3D micro livers which were capable of stably maintaining metabolism of essential materials like glucose, lipids, and drugs, as well as production and secretion of bile. Using the Regenova technology (not to be confused with Chinese Regenovo) designed by Cyfuse, a Japanese biomedical company, liver bioprinting was undertaken using the Kenzan method. Developed by Professor Koich Nakayama and Cyfuse Biomedical, the Kenzan method is a scaffold-free method which eliminates the need for supporting scaffolds. By removing the scaffolds, cell functionality could be extended as cells were not dependant on any outside support from a scaffold. The liver structures formed using this method exhibited self-organisation, ECM production, and a wide array of other metabolic functions that could be maintained over long periods. They anticipate that this unique liver model may become widely used in drug discovery studies, and may assist with the identification of new drug targets, safety studies, and pharmacokinetics [261].

### Advanced 3D cell culture models: a regulatory perspective

With these promising advances observed worldwide, regulators are moving into the 3D space in order to accelerate drug discovery. In the USA, the NIH has a program which develops bioprinted tissues, in collaboration with Organovo, with the goal of speeding up drug screening. NCATS, or the National Centre for Advancing Translational Sciences, acknowledges that the current methods for developing and delivering drugs to patients takes decades, incurs huge costs up to billions, and approximately 95% of the time are unsuccessful, and therefore has launched a 3D tissue bioprinting program, combining living cells and scaffolds to test materials in platforms that closely mimic human tissues and organs in order to better predict human responses to potential therapies [262]. NCATS has also developed “on-chip” technology with their Tissue Chip for Drug Screening initiative [263]. It can be noted however that despite the work done in NCATS and other institutions, many challenges still remain to successfully construct various 3D bioprinting generated functional organs and actually apply them to pre-clinical assessment and regenerative medicine. This is particularly true with regard to obtaining FDA approval, and despite having moderate success with producing candidates for rare diseases that have been picked up by pharma and which have reached clinical trials, the only FDA approval it has achieved was in 2015 when it showed that the drug lymphangioleiomyomatosis (LAM), previously used to prevent organ rejection, could be used to treat a rare lung disease. Dr Chris Austin, a neurologist at the NIH and director of NCATS, acknowledges that many roadblocks exist to drug development, and include the fact that advanced methodologies for pre-clinical assessment such as “on-chip” technology, induced pluripotent stem cells, and 3D bioprinted organs have yet to be widely accepted and adopted by drug companies as in vitro alternatives to conventional animal tests, and that the pharma industry is “conservative” and slow to embrace new technologies [264]. A key issue can also be seen in the failure of the FDA to lay out specific pathways for accepting these new technologies in lieu of animal experimentation [265]. One success can be observed in the orphan drug designation Organovo received in 2017 from the FDA for their 3D bioprinted liver tissues used in the treatment of alpha-1 antitrypsin (A1AT) deficiency [264]. Following this designation however, another key issue became apparent, the huge costs incurred from 3D bioprinting, and despite Organovo anticipating that by the end of 2019 they would have two liver tissues on track for 2020, increased costs and extended timelines have pushed human testing back to 2021.

Despite the future looking promising from both a technical and scientific perspective regarding these advanced technologies, many regulatory hurdles surrounding them exist. One such hurdle is the classification of tissues and organs bioprinted using 3D methods. Should a 3D printed liver be classed as an organ or a product? And with regard to regulation, is it a medical device? These are questions that regulators must answer in order to advance this area, and decisions need to be made as to whether new regulations should be developed for bioprinting or should they be covered under existing frameworks. Some commentators in the EU and the USA have also raised the issue of morality surrounding bioprinting tissues and organs and have questioned whether bioprinted materials should fall under patent protection, reminiscent of the Dolly the sheep case [266]. A study at Bournemouth University and funded by the EC published in April 2020 has assisted these issues and provides legal guidance on the regulatory and intellectual property issues surrounding 3D bioprinting technology, and it is acknowledged today that if EU regulatory classifies bioprinted products as medical devices, some degree of clarity will exist legally as guidelines for medical devices have been in place for decades. In the USA, the FDA has issued guidelines on 3D bioprinted medical devices themselves, but not on the specifics of their printing. These guidelines are also not legally binding, meaning ambiguity still exists. These is no doubt that the era of 3D bioprinting will significantly advance modern medicine and the lives of countless patients; however, for this to happen, policymakers and regulators must ensure that the correct guidelines and regulations are in place. However, optimisation must be undertaken before they can be implemented widely as pre-clinical assessment tools for NBMs or other materials (e.g., active pharmaceutical ingredients, health technology, or theranostics). To date, no standardised or validated 3D model, either a healthy or diseased state, is approved for pre-clinical screening of NBM liver toxicity at the regulatory level. Hence, the need for alternative models which can be successfully integrated into the NBM screening pipeline is still unmet.

## Conclusions

In conclusion, the vast array of applications for NBMs have made them extremely beneficial in many fields, and they offer many exciting opportunities in medicine; however, despite their advantages, their translation to the clinic is overwhelmingly slow and despite great investment, in recent decades only a small number of NBM-based products have been translated into viable medical products [1]. This lack of translation and indeed high attrition rate is in large part due to inappropriate pre-clinical assessment screening methodologies for testing NBM toxicity, in particular hepatotoxicity. Whilst conventional 2D in vitro monoculture liver models formed from immortalised or transformed cell lines are long established and widely used due to their distinct advantages including lack of donor variation and resistance to senescence, they have reduced metabolic capacities, altered phenotypes, loss of polarity and contact inhibition, and a decrease in liver-specific function, among other key failings. In vitro models also fall short in predicting toxicity due to issues such as non-organ-specific toxicity, non-linear dose–toxicity, unclear mechanisms, lack of the key structural and functional characteristics previously detailed and which include hepatic zonation, secondary structure formation, and in vivo-like cell heterogenicity, and hepatocyte polarity. Whilst some of these issues may be overcome by using PHH, supply of these cells is limited; inter-donor variation and difficulties in maintaining a stable phenotype due to the 2D culture environment could be other limiting factors [74–78]. A further way to overcome key hurdles seen with conventional liver cultures, the culture of multiple liver cell types in co-culture models, does allow some advantages over monocultures; however, there are limitations on how many cell lines can be cultured together, great variability is observed, and there is a distinct lack of ECM components, all of which impact the cells response to NBMs.

On the other arm of in vivo liver toxicity screening methods are animal models, used to provide a basic overview of the fate of NBMs in organs, and to provide information on dosing and potential toxicities. In vivo animal models for the screening of NBMs are most often undertaken in porcine or rodent models [96], in accordance with the European Commission Directive 2010/63/EU, and today they are regarded as an essential component in the translation of NBMs from lab to clinic. Nevertheless, in a similar manner to in vitro methods, they are not without the own intrinsic limitations, and whilst rodent models incur low costs, are small and easy to handle, reproduce fast, and bear close resemblance to humans in terms of genetics and biology, there are fundamental differences between human and rodent anatomy, physiology, and immune response [110–112, 267]. Whilst porcine models are useful in the pre-clinical assessment of NBMs due to the presence of human-specific cell types which cannot be found in rodents [116], and their more similar physical size and similarities to humans with regard to physiology, anatomy, epigenetics, and immunogenetics, porcine models are costly, and one must wonder why if animal studies are so predictive of human toxicity are drug and NBM attrition rates still so high? Today consensus among many scientists is that in vivo animal models are not as accurate at predicting human response as we may have previously assumed [148, 149], particularly regarding hepatotoxicity [152–154]. These issues, coupled with the need for newer, more bio-comparable assessment models for predicting patient outcomes are what has, in recent years, driven the development of 3D methodologies which may act as a bridge between conventional 2D and in vivo.

These 3D methodologies are now seen as vitally important emerging technologies in the NBM pre-clinical assessment cascade [11], and in recent years, sophisticated, physiologically relevant 3D liver models that can more accurately predict the hepatotoxicity of NBMs have been developed [173]. These models not only recapitulate whole organ physiology, but are also fit for repeated exposures and chronic drug testing, an important consideration with regard to NBM pre-clinical assessment [19]. These 3D cell culture models, namely cell spheroids and MCTS, scaffold and sandwich cultures, organoids, ex vivo models, and more complex models such as MPS, “on-chip”, and bioprinting technologies may act as huge time- and cost-savers in the NBM development pipeline, overcoming some of the key challenges faced in NBM assessment, provided the appropriate validation is undertaken and their efficacy in pre-clinical testing is proven.

From the work undertaken at the Max Planck Institute [257], to Organovo’s bioprinting [132, 190], advanced liver models are set to revolutionise drug and NBM safety screening, with funding agencies and regulators now paying close attention to the potential of these advanced technologies, something which is reflected in the multiple large-scale Horizon 2020–funded EU projects that have had many successful outcomes to date with regard to advanced liver models for NBM toxicity screening [250–256]. In the USA, the Wyss Institute is at the forefront of material and device bioprinting with their SWIFT technology, which is anticipated to be a silver bullet for resolving organ shortages worldwide [268]. Utilising “organ-on-chip” technology, the Wyss Institute has also successfully reproduced many complex organ-level responses in no less than ten different organ-on-chip models [269], and has also developed a device to link them, in a “body-on-chip” model [270]. Many of its innovations have also been commercially translated, with financial support obtained from many different government agencies, and it also works in partnership with various major pharmaceutical companies [249]. Further afield, countries like Brazil, Japan, and China have also made great progress with the development of advanced hepatic models and 3D bioprinting technology [258–261]. The scientific culture within the biomedical field has certainly shifted in recent years, and animal experimentation and 2D in vitro pre-clinical assessment are not as immune to controversy and criticism as they once were. This shift is associated with the fact that, in recent years 3D models have been shown to be vastly more beneficial than conventional pre-clinical assessment methods in NBM screening, with the intrinsic limitations of conventional in vitro and in vivo methods as the primary motivators for the development and validation of these new emerging 3D technologies. These physiologically relevant and advanced in vitro systems have the capacity to increase relevance of routine cell cultures whilst overcoming the interspecies difference characteristics of in vivo studies, thus becoming valuable tools for understanding the mechanisms of both drug and NBM toxicity. The ability to assess the fate and toxicity of NBMs in the liver in a human-relevant manner during their pre-clinical assessment is a huge advantage. Despite all of these advances however, many roadblocks still exist, and even though there is a strong need for the availability of new, more predictive, and ethical methodologies for NBM pre-clinical assessment, it may be many years until researchers, stakeholders, funding agencies, and governments fully accept this change and make critical moves towards implementing these new methodologies. Thus, future efforts in the biomedical scientific community should aim at validating existing advanced methodologies and obtaining regulatory approval (e.g., OECD certification, EMA/FDA acceptance), an official recognition of the predictive value of an advanced 3D methodology, a factor that will allow their widespread implementation across academia as well as industry.

## Data Availability

N/A.

## References

[1] Marques MRC (2019). Nanomedicines - tiny particles and big challenges. Adv Drug Deliv Rev.

[2] Nanomedicine and the COVID-19 vaccines*.* Nature Nanotechnology. 2020;15(12):963–963. 10.1038/s41565-020-00820-0 PMC7692425 33247210

[3] van der Meel R, Lammers T, Hennink WE (2017). Cancer nanomedicines: oversold or underappreciated?. Expert Opin Drug Deliv.

[4] Soares S, et al. Nanomedicine: principles, properties, and regulatory issues*.* Front Chem. 2018;6(360). 10.3389/fchem.2018.00360 PMC6109690 30177965

[5] Wolfram J (2015). Safety of Nanoparticles in Medicine. Curr Drug Targets.

[6] Lee WM (2003). Drug-induced hepatotoxicity. N Engl J Med.

[7] Wu T, Tang M (2018). Review of the effects of manufactured nanoparticles on mammalian target organs. J Appl Toxicol.

[8] Zhao L, Zhang B (2017). Doxorubicin induces cardiotoxicity through upregulation of death receptors mediated apoptosis in cardiomyocytes. Sci Rep.

[9] Gabizon A (1994). Clinical studies of liposome-encapsulated doxorubicin. Acta Oncol.

[10] Hengge UR (1993). Fatal hepatic failure with liposomal doxorubicin. Lancet.

[11] Siegrist S (2019). Preclinical hazard evaluation strategy for nanomedicines. Nanotoxicology.

[12] Etheridge ML (2013). The big picture on nanomedicine: the state of investigational and approved nanomedicine products. Nanomedicine.

[13] Pelaz B (2017). Diverse applications of nanomedicine. ACS Nano.

[14] Collins SD, Yuen G, Tu T, et al. In vitro models of the liver: disease modeling, drug discovery and clinical applications*.*, in Hepatocellular carcinoma. T.-P. JEE, Editor. 2009. 31664801

[15] Fröhlich E (2018). Comparison of conventional and advanced in vitro models in the toxicity testing of nanoparticles. Artif Cells Nanomed Biotechnol.

[16] Kumar V, Sharma N, Maitra SS (2017). In vitro and in vivo toxicity assessment of nanoparticles. International Nano Letters.

[17] Jensen C, Teng Y. Is it time to start transitioning from 2D to 3D cell culture?. Front Molecular Biosci. 2020:7(33). 10.3389/fmolb.2020.00033 PMC7067892 32211418

[18] Collins SD, et al. In vitro models of the liver: disease modeling, drug discovery and clinical applications, in Hepatocellular carcinoma. J.E.E. Tirnitz-Parker, Editor. 2019, Codon Publications Brisbane (AU). 2019;48–60. 31664801

[19] Zhou Y, Shen JX, Lauschke VM. Comprehensive evaluation of organotypic and microphysiological liver models for prediction of drug-induced liver injury*.* 2019;10(1093). 10.3389/fphar.2019.01093 PMC6769037 31616302

[20] Soldatow VY (2013). In vitro models for liver toxicity testing. Toxicol Res (Camb).

[21] Abdel-Misih SR, Bloomston M (2010). Liver anatomy. Surg Clin North Am.

[22] Vigue J. Asklepios atlas of human anatomy. 2014.

[23] Godoy P (2013). Recent advances in 2D and 3D in vitro systems using primary hepatocytes, alternative hepatocyte sources and non-parenchymal liver cells and their use in investigating mechanisms of hepatotoxicity, cell signaling and ADME. Arch Toxicol.

[24] Bacon BR, O'Grady JG, Di Bisceglie AM, Lake JR. Comprehensive clinical hepatology. Elsevier Ltd; 2006.

[25] Portmann BC. Chapter 1 - Development and anatomy of the normal liver, in Comprehensive clinical hepatology (second edition), b.R. Bacon, et al., Editors. 2006, Mosby: Edinburgh. p. 1–15.

[26] Sun T (2014). Engineered nanoparticles for drug delivery in cancer therapy. Angew Chem Int Ed Engl.

[27] Zhang Y-N (2016). Nanoparticle–liver interactions: cellular uptake and hepatobiliary elimination. J Control Release.

[28] Gissen P, Arias IM (2015). Structural and functional hepatocyte polarity and liver disease. J Hepatol.

[29] Soars MG (2007). The pivotal role of hepatocytes in drug discovery. Chem Biol Interact.

[30] Zhou Z, Xu MJ, Gao B (2016). Hepatocytes: a key cell type for innate immunity. Cell Mol Immunol.

[31] Friedman LS. Diseases of the liver, seventh edition. Edited by L. Schiff and E. R. Schiff, 1,516 pp. Philadelphia: J.B. Lippincott*,* 1993. $195*.* Hepatology. 1994;19(3):797–798.

[32] Haute DV, Berlin JM (2017). Challenges in realizing selectivity for nanoparticle biodistribution and clearance: lessons from gold nanoparticles. Ther Deliv.

[33] Saha K (2016). Regulation of macrophage recognition through the interplay of nanoparticle surface functionality and protein corona. ACS Nano.

[34] Smith DA (2019). Clearance in drug design. J Med Chem.

[35] Zhang YN (2016). Nanoparticle-liver interactions: cellular uptake and hepatobiliary elimination. J Control Release.

[36] Samuelsson E (2017). Contribution of Kupffer cells to liposome accumulation in the liver. Colloids Surf, B.

[37] Champion JA, Mitragotri S (2009). Shape induced inhibition of phagocytosis of polymer particles. Pharm Res.

[38] He C (2010). Effects of particle size and surface charge on cellular uptake and biodistribution of polymeric nanoparticles. Biomaterials.

[39] Lunov O (2011). Modeling receptor-mediated endocytosis of polymer-functionalized iron oxide nanoparticles by human macrophages. Biomaterials.

[40] Kamps JAAM (1997). Massive targeting of liposomes, surface-modified with anionized albumins, to hepatic endothelial cells. Proc Natl Acad Sci.

[41] Adrian JE (2007). Delivery of viral vectors to hepatic stellate cells in fibrotic livers using HVJ envelopes fused with targeted liposomes. J Drug Target.

[42] Adrian JE (2007). Effects of a new bioactive lipid-based drug carrier on cultured hepatic stellate cells and liver fibrosis in bile duct-ligated rats. J Pharmacol Exp Ther.

[43] Du S-L (2007). Cyclic Arg-Gly-Asp peptide-labeled liposomes for targeting drug therapy of hepatic fibrosis in rats. J Pharmacol Exp Ther.

[44] Mezghrani O (2015). Hepatocellular carcinoma dually-targeted nanoparticles for reduction triggered intracellular delivery of doxorubicin. Int J Pharm.

[45] Sato Y (2008). Resolution of liver cirrhosis using vitamin A-coupled liposomes to deliver siRNA against a collagen-specific chaperone. Nat Biotechnol.

[46] Akhter A (2014). Ligand density at the surface of a nanoparticle and different uptake mechanism: two important factors for successful siRNA delivery to liver endothelial cells. Int J Pharm.

[47] Kren BT (2009). Nanocapsule-delivered Sleeping Beauty mediates therapeutic factor VIII expression in liver sinusoidal endothelial cells of hemophilia A mice. J Clin Invest.

[48] LeCluyse EL, Audus KL, Hochman JH (1994). Formation of extensive canalicular networks by rat hepatocytes cultured in collagen-sandwich configuration. Am J Physiol.

[49] Choi JM (2015). HepG2 cells as an in vitro model for evaluation of cytochrome P450 induction by xenobiotics. Arch Pharm Res.

[50] Elizondo G, Medina-Diaz IM (2003). Induction of CYP3A4 by 1alpha,25-dyhydroxyvitamin D3 in HepG2 cells. Life Sci.

[51] Liu MC (1985). Tyrosine sulfation of proteins from the human hepatoma cell line HepG2. Proc Natl Acad Sci U S A.

[52] Vermeir M (2005). Cell-based models to study hepatic drug metabolism and enzyme induction in humans. Expert Opin Drug Metab Toxicol.

[53] Kermanizadeh A (2012). An in vitro liver model - assessing oxidative stress and genotoxicity following exposure of hepatocytes to a panel of engineered nanomaterials. Part Fibre Toxicol.

[54] Bandele OJ (2012). In vitro toxicity screening of chemical mixtures using HepG2/C3A cells. Food Chem Toxicol.

[55] Bale SS (2014). In vitro platforms for evaluating liver toxicity. Exp Biol Med (Maywood).

[56] Nibourg GA (2012). Proliferative human cell sources applied as biocomponent in bioartificial livers: a review. Expert Opin Biol Ther.

[57] Guillouzo A (2007). The human hepatoma HepaRG cells: a highly differentiated model for studies of liver metabolism and toxicity of xenobiotics. Chem Biol Interact.

[58] Donato MT (2008). Cell lines: a tool for in vitro drug metabolism studies. Curr Drug Metab.

[59] Gerets HH (2012). Characterization of primary human hepatocytes, HepG2 cells, and HepaRG cells at the mRNA level and CYP activity in response to inducers and their predictivity for the detection of human hepatotoxins. Cell Biol Toxicol.

[60] O'Brien PJ (2006). High concordance of drug-induced human hepatotoxicity with in vitro cytotoxicity measured in a novel cell-based model using high content screening. Arch Toxicol.

[61] Zeilinger K (2016). Cell sources for in vitro human liver cell culture models. Exp Biol Med (Maywood).

[62] Huang JR, et al. Liposomal irinotecan for treatment of colorectal cancer in a preclinical model*.* Cancers (Basel). 2019;11(3). 10.3390/cancers11030281 PMC6468623 30818855

[63] Wang P (2018). Evaluating cellular uptake of gold nanoparticles in HL-7702 and HepG2 cells for plasmonic photothermal therapy. Nanomedicine.

[64] Rathinaraj P (2015). Targeting and molecular imaging of HepG2 cells using surface-functionalized gold nanoparticles. J Nanopart Res.

[65] Ashokkumar T (2014). Apoptosis in liver cancer (HepG2) cells induced by functionalized gold nanoparticles. Colloids Surf B Biointerfaces.

[66] Namvar F (2014). Cytotoxic effect of magnetic iron oxide nanoparticles synthesized via seaweed aqueous extract. Int J Nanomedicine.

[67] Sulheim E, et al. Cytotoxicity of poly(alkyl cyanoacrylate) nanoparticles*.* Int J Mol Sci. 2017;18(11). 10.3390/ijms18112454 PMC5713421 29156588

[68] Sulheim E (2016). Cellular uptake and intracellular degradation of poly(alkyl cyanoacrylate) nanoparticles. J Nanobiotechnol.

[69] Ramboer E (2015). Immortalized human hepatic cell lines for in vitro testing and research purposes. Methods Mol Biol.

[70] Prozialeck WC (2006). Epithelial barrier characteristics and expression of cell adhesion molecules in proximal tubule-derived cell lines commonly used for in vitro toxicity studies. Toxicol In Vitro.

[71] Chamberlain LM (2009). Phenotypic non-equivalence of murine (monocyte-) macrophage cells in biomaterial and inflammatory models. J Biomed Mater Res A.

[72] Milyavsky M (2003). Prolonged culture of telomerase-immortalized human fibroblasts leads to a premalignant phenotype. Cancer Res.

[73] Ramboer E, et al. Immortalized human hepatic cell lines for in vitro testing and research purposes*.* Methods in molecular biol (Clifton, N.J.). 2015;1250:53–76. 10.1007/978-1-4939-2074-7_4 PMC4579543 26272134

[74] Ponsoda X, et al. Drug biotransformation by human hepatocytes. In vitro/in vivo metabolism by cells from the same donor*.* J Hepatol. 2001;34(1):19–25. 10.1016/s0168-8278(00)00085-4 11211902

[75] Gomez-Lechon MJ (2003). Human hepatocytes as a tool for studying toxicity and drug metabolism. Curr Drug Metab.

[76] Gomez-Lechon MJ (2004). Human hepatocytes in primary culture: the choice to investigate drug metabolism in man. Curr Drug Metab.

[77] Knobeloch D (2012). Human hepatocytes: isolation, culture, and quality procedures. Methods Mol Biol.

[78] Rodriguez-Antona C (2002). Cytochrome P450 expression in human hepatocytes and hepatoma cell lines: molecular mechanisms that determine lower expression in cultured cells. Xenobiotica.

[79] Hartung T, Daston G (2009). Are in vitro tests suitable for regulatory use?. Toxicol Sci.

[80] Granitzny A (2017). Evaluation of a human in vitro hepatocyte-NPC co-culture model for the prediction of idiosyncratic drug-induced liver injury: A pilot study. Toxicol Rep.

[81] Bale SS (2016). Isolation and co-culture of rat parenchymal and non-parenchymal liver cells to evaluate cellular interactions and response. Sci Rep.

[82] Ha S-W, et al. Chapter 4 - Applications of silica-based nanomaterials in dental and skeletal biology, in Nanobiomaterials in clinical dentistry (second edition), K. Subramani and W. Ahmed, Editors. 2019, Elsevier. p. 77–112.

[83] Bale SS (2015). Long-term coculture strategies for primary hepatocytes and liver sinusoidal endothelial cells. Tissue Eng Part C Methods.

[84] Ohno M (2009). Induction of drug-metabolizing enzymes by phenobarbital in layered co-culture of a human liver cell line and endothelial cells. Biol Pharm Bull.

[85] Adams DH (2010). Mechanisms of immune-mediated liver injury. Toxicol Sci.

[86] West MA (1986). Further characterization of Kupffer cell/macrophage-mediated alterations in hepatocyte protein synthesis. Surgery.

[87] Yagi K (1998). Stimulation of liver functions in hierarchical co-culture of bone marrow cells and hepatocytes. Cytotechnology.

[88] Ha S-W, et al. Applications of silica-based nanomaterials in dental and skeletal biology, in Nanobiomaterials in clinical dentistry, K. Subramani and W. Ahmed, Editors. Elsevier. 2019;77–112.

[89] Edling Y (2009). Increased sensitivity for troglitazone-induced cytotoxicity using a human in vitro co-culture model. Toxicol In Vitro.

[90] Esch MB (2014). Body-on-a-chip simulation with gastrointestinal tract and liver tissues suggests that ingested nanoparticles have the potential to cause liver injury. Lab Chip.

[91] Duval K, et al. Modeling physiological events in 2D vs. 3D cell culture*.* Physiol (Bethesda). 2017;32(4):266–277. 10.1152/physiol.00036.2016 PMC5545611 28615311

[92] Olsavsky Goyak KM, Laurenzana EM, Omiecinski CJ. Hepatocyte differentiation*.* Methods Mol Biol. 2010;640:115–38. 10.1007/978-1-60761-688-7_6 PMC6690360 20645049

[93] Kapalczynska M (2018). 2D and 3D cell cultures - a comparison of different types of cancer cell cultures. Arch Med Sci.

[94] Gomez-Lechon MJ (1998). Long-term expression of differentiated functions in hepatocytes cultured in three-dimensional collagen matrix. J Cell Physiol.

[95] Wells RG (2008). The role of matrix stiffness in regulating cell behavior. Hepatology.

[96] Mitragotri S (2017). Drug delivery research for the future: expanding the nano horizons and beyond. J Control Release.

[97] Bryda EC (2013). The Mighty Mouse: the impact of rodents on advances in biomedical research. Mo Med.

[98] Bahamonde J (2018). Gold nanoparticle toxicity in mice and rats: species differences. Toxicol Pathol.

[99] Lu WL (2004). A pegylated liposomal platform: pharmacokinetics, pharmacodynamics, and toxicity in mice using doxorubicin as a model drug. J Pharmacol Sci.

[100] Recordati C (2016). Tissue distribution and acute toxicity of silver after single intravenous administration in mice: nano-specific and size-dependent effects. Part Fibre Toxicol.

[101] Chen Z (2019). Hepatotoxicity and the role of the gut-liver axis in rats after oral administration of titanium dioxide nanoparticles. Part Fibre Toxicol.

[102] Tang H (2019). Liver toxicity assessments in rats following sub-chronic oral exposure to copper nanoparticles. Environ Sci Eur.

[103] Khan HA, et al. Effects of naked gold nanoparticles on proinflammatory cytokines mRNA expression in rat liver and kidney*.* BioMed Res Int. 2013;2013:590730. 10.1155/2013/590730 PMC3677657 23781503

[104] Abdelhalim MAK. Uptake of gold nanoparticles in several rat organs after intraperitoneal administration in vivo: a fluorescence study*.* BioMed Res Int. 2013;2013:353695. 10.1155/2013/353695 PMC3730147 23956977

[105] Abdelhalim MAK, Abdelmottaleb Moussa SA. The gold nanoparticle size and exposure duration effect on the liver and kidney function of rats: in vivo*.* Saudi J Biol Sci. 2013;20(2):177–181. 10.1016/j.sjbs.2013.01.007 PMC3730801 23961234

[106] Abdelhalim MAK, Mady MM (2011). Liver uptake of gold nanoparticles after intraperitoneal administration in vivo: a fluorescence study. Lipids Health Dis.

[107] Yahyaei B (2019). Effects of biologically produced gold nanoparticles: toxicity assessment in different rat organs after intraperitoneal injection. AMB Express.

[108] Kozics K, et al. Pharmacokinetics, biodistribution, and biosafety of PEGylated gold nanoparticles in vivo*.* Nanomaterials (Basel). 2021;11(7). 10.3390/nano11071702 PMC8305691 34203551

[109] Gaspar MM (2012). Targeted delivery of transferrin-conjugated liposomes to an orthotopic model of lung cancer in nude rats. J Aerosol Med Pulm Drug Deliv.

[110] Le Magnen C, Dutta A, Abate-Shen C (2016). Optimizing mouse models for precision cancer prevention. Nat Rev Cancer.

[111] Begley CG, Ellis LM (2012). Drug development: raise standards for preclinical cancer research. Nature.

[112] Cook N, Jodrell DI, Tuveson DA (2012). Predictive in vivo animal models and translation to clinical trials. Drug Discov Today.

[113] Seok J (2013). Genomic responses in mouse models poorly mimic human inflammatory diseases. Proc Natl Acad Sci U S A.

[114] de Souza N (2013). Mouse models challenged. Nat Methods.

[115] Rangarajan A, Weinberg RA (2003). Comparative biology of mouse versus human cells: modelling human cancer in mice. Nat Rev Cancer.

[116] Schachtschneider KM (2017). A validated, transitional and translational porcine model of hepatocellular carcinoma. Oncotarget.

[117] Ribitsch I, et al. Large animal models in regenerative medicine and tissue engineering: to do or not to do*.* Front Bioeng Biotechnol. 2020;8. 10.3389/fbioe.2020.00972 PMC7438731 32903631

[118] Andrasina T (2019). The accumulation and effects of liposomal doxorubicin in tissues treated by radiofrequency ablation and irreversible electroporation in liver: in vivo experimental study on porcine models. Cardiovasc Intervent Radiol.

[119] Edge D (2016). Pharmacokinetics and bio-distribution of novel super paramagnetic iron oxide nanoparticles (SPIONs) in the anaesthetized pig. Clin Exp Pharmacol Physiol.

[120] Ungureanu BS (2015). Iron oxide nanoparticles biodistribution in an experimental pig model - a new approach for delivery and imaging. Curr Health Sci J.

[121] Kociova S (2020). Zinc phosphate-based nanoparticles as alternatives to zinc oxide in diet of weaned piglets. Journal of Animal Science and Biotechnology.

[122] Saleh T (2018). Silver nanoparticles improve structural stability and biocompatibility of decellularized porcine liver. Artificial Cells, Nanomedicine, and Biotechnology.

[123] Hannon G (2019). Immunotoxicity considerations for next generation cancer nanomedicines. Advanced Science.

[124] Szebeni J (2018). Roadmap and strategy for overcoming infusion reactions to nanomedicines. Nat Nanotechnol.

[125] Vu MN, et al. Current and future nanoparticle vaccines for COVID-19*.* eBioMedicine. 2021;74:103699. 10.1016/j.ebiom.2021.103699 PMC8602808 34801965

[126] Sieber S (2019). Zebrafish as a preclinical in vivo screening model for nanomedicines. Adv Drug Deliv Rev.

[127] Rennekamp AJ, Peterson RT (2015). 15 years of zebrafish chemical screening. Curr Opin Chem Biol.

[128] Peterson RT (2000). Small molecule developmental screens reveal the logic and timing of vertebrate development. Proc Natl Acad Sci U S A.

[129] Sieber S (2017). Zebrafish as an early stage screening tool to study the systemic circulation of nanoparticulate drug delivery systems in vivo. J Control Release.

[130] EC. Directive 2010/63/EU of the European Parliament and of the Council of 22 September 2020 on the Protection of Animals Used for Scientific Purposes*.* 2010.

[131] Goldsmith P (2004). Zebrafish as a pharmacological tool: the how, why and when. Curr Opin Pharmacol.

[132] Menke AL (2011). Normal anatomy and histology of the adult zebrafish.

[133] Jung HM (2017). Development of the larval lymphatic system in zebrafish. Development.

[134] Chen AT, Zon LI (2009). Zebrafish blood stem cells. J Cell Biochem.

[135] Trede NS (2004). The use of zebrafish to understand immunity. Immunity.

[136] Isogai S, Horiguchi M, Weinstein BM (2001). The vascular anatomy of the developing zebrafish: an atlas of embryonic and early larval development. Dev Biol.

[137] Chu J, Sadler KC. New school in liver development: lessons from zebrafish*.* Hepatol (Baltimore, Md.). 2009;50(5):1656–1663. 10.1002/hep.23157 PMC3093159 19693947

[138] Howe K (2013). The zebrafish reference genome sequence and its relationship to the human genome. Nature.

[139] Vibe CB (2016). Thioridazine in PLGA nanoparticles reduces toxicity and improves rifampicin therapy against mycobacterial infection in zebrafish. Nanotoxicology.

[140] Peng K (2010). Cyclodextrin/dextran based drug carriers for a controlled release of hydrophobic drugs in zebrafish embryos. Soft Matter.

[141] Yan H (2012). Functional mesoporous silica nanoparticles for photothermal-controlled drug delivery in vivo.

[142] Hason M, Bartůněk P. Zebrafish models of cancer-new insights on modeling human cancer in a non-mammalian vertebrate*.* Genes (Basel). 2019;10(11). 10.3390/genes10110935 PMC6896156 31731811

[143] Holbech H (2006). Detection of endocrine disrupters: evaluation of a fish sexual development test (FSDT). Comp Biochem Physiol C Toxicol Pharmacol.

[144] Fomchenko EI, Holland EC (2006). Mouse models of brain tumors and their applications in preclinical trials. Clin Cancer Res.

[145] Huff J, Jacobson MF, Davis DL (2008). The limits of two-year bioassay exposure regimens for identifying chemical carcinogens. Environ Health Perspect.

[146] Van Norman GA (2019). Limitations of animal studies for predicting toxicity in clinical trials: is it time to rethink our current approach?. JACC Basic Transl Sci.

[147] Van Norman GA. Drugs, devices, and the FDA: Part 1: An overview of approval processes for drugs*.* JACC: Basic to Translational Sci. 2016;1(3):170–179. 10.1016/j.jacbts.2016.03.002 PMC6113160 30167510

[148] Shanks N, Greek R, Greek J (2009). Are animal models predictive for humans?. Philosophy, ethics, and humanities in medicine : PEHM.

[149] Greek R, Menache A (2013). Systematic reviews of animal models: methodology versus epistemology. Int J Med Sci.

[150] Hackam DG, Redelmeier DA (2006). Translation of research evidence from animals to humans. JAMA.

[151] Perel P (2007). Comparison of treatment effects between animal experiments and clinical trials: systematic review. BMJ.

[152] Olson H (2000). Concordance of the toxicity of pharmaceuticals in humans and in animals. Regul Toxicol Pharmacol.

[153] Pound P (2004). Where is the evidence that animal research benefits humans?. BMJ.

[154] Bracken MB (2009). Why animal studies are often poor predictors of human reactions to exposure. J R Soc Med.

[155] Xu JJ, Diaz D, O'Brien PJ (2004). Applications of cytotoxicity assays and pre-lethal mechanistic assays for assessment of human hepatotoxicity potential. Chem Biol Interact.

[156] McKenzie R (1995). Hepatic failure and lactic acidosis due to fialuridine (FIAU), an investigational nucleoside analogue for chronic hepatitis B. N Engl J Med.

[157] Institute of Medicine Committee to Review the Fialuridine Clinical T. In Review of the fialuridine (FIAU) clinical trials, F.J. Manning and M. Swartz, Editors. National Academies Press (US): Washington (DC). 1995. 25121268

[158] Attarwala H (2010). TGN1412: from discovery to disaster. J Young Pharm.

[159] Xu D, et al. Fialuridine induces acute liver failure in chimeric TK-NOG mice: a model for detecting hepatic drug toxicity prior to human testing*.* PLoS Med. 2014;11(4):e1001628. 10.1371/journal.pmed.1001628 PMC3988005 24736310

[160] Babai S, Auclert L, Le-Louët H. Safety data and withdrawal of hepatotoxic drugs*.* Therapie. 2018. 10.1016/j.therap.2018.02.004 29609830

[161] Villano JL, Mehta D, Radhakrishnan L (2006). Abraxane induced life-threatening toxicities with metastatic breast cancer and hepatic insufficiency. Invest New Drugs.

[162] Socinski M (2006). Update on nanoparticle albumin-bound paclitaxel. Clin Adv Hematol Oncol.

[163] Administration USFaD. Investigational new drug (IND) application. U.S. Food and Drug Administration*.* 2017.

[164] Zhang D (2012). Preclinical experimental models of drug metabolism and disposition in drug discovery and development. Acta Pharmaceutica Sinica B.

[165] Akhtar A (2015). The flaws and human harms of animal experimentation. Camb Q Healthc Ethics.

[166] Di Cristo L, et al. Towards the identification of an in vitro tool for assessing the biological behavior of aerosol supplied nanomaterials*.* Int J Environ Res Public Health. 2018;15(4). 10.3390/ijerph15040563 PMC5923605 29561767

[167] Bregoli L (2016). Nanomedicine applied to translational oncology: a future perspective on cancer treatment. Nanomedicine.

[168] Movia D (2014). A safe-by-design approach to the development of gold nanoboxes as carriers for internalization into cancer cells. Biomaterials.

[169] Movia D, Bruni-Favier S, Prina-Mello A (2020). In vitro alternatives to acute inhalation toxicity studies in animal models-a perspective. Frontiers in bioengineering and biotechnology.

[170] Movia D, Prina-Mello A. Preclinical development of orally inhaled drugs (OIDs)-are animal models predictive or shall we move towards in vitro non-animal models?. Animals (Basel). 2020;10(8). 10.3390/ani10081259 PMC7460012 32722259

[171] Prina-Mello A, et al. Editorial: Use of 3D models in drug development and precision medicine - advances and outlook*.* Front Bioeng Biotechnol. 2021;9(137). 10.3389/fbioe.2021.658941 PMC7965946 33748095

[172] Prina-Mello DMaA. Nanotoxicity in cancer research: technical protocols and considerations for the use of 3D tumour spheroids, unraveling the safety profile of nanoscale particles and materials, in From biomedical to environmental applications. IntechOpen. 2017.

[173] Alepee N (2014). State-of-the-art of 3D cultures (organs-on-a-chip) in safety testing and pathophysiology. Altex.

[174] Fleddermann J (2019). Distribution of SiO2 nanoparticles in 3D liver microtissues. Int J Nanomedicine.

[175] Ozkan A (2019). In vitro vascularized liver and tumor tissue microenvironments on a chip for dynamic determination of nanoparticle transport and toxicity. Biotechnol Bioeng.

[176] Otieno MA, Gan J, Proctor W. Status and future of 3D cell culture in toxicity testing, in Drug-induced liver toxicity, M. Chen and Y. Will, Editors. Springer New York: New York, NY. 2018;249–261.

[177] Duval K, et al. Modeling physiological events in 2D vs. 3D cell culture*.* Physiol (Bethesda, Md.). 2017;32(4):266–277. 10.1152/physiol.00036.2016 PMC5545611 28615311

[178] Fang Y, Eglen RM (2017). Three-dimensional cell cultures in drug discovery and development. SLAS Discov.

[179] Deng J (2019). Engineered liver-on-a-chip platform to mimic liver functions and its biomedical applications: a review. Micromachines (Basel).

[180] Dunn JC, Tompkins RG, Yarmush ML (1992). Hepatocytes in collagen sandwich: evidence for transcriptional and translational regulation. J Cell Biol.

[181] Dunn JC, Tompkins RG, Yarmush ML (1991). Long-term in vitro function of adult hepatocytes in a collagen sandwich configuration. Biotechnol Prog.

[182] Dunn JC (1989). Hepatocyte function and extracellular matrix geometry: long-term culture in a sandwich configuration. FASEB J.

[183] Molina-Jimenez F (2012). Matrigel-embedded 3D culture of Huh-7 cells as a hepatocyte-like polarized system to study hepatitis C virus cycle. Virology.

[184] Swift B, Pfeifer ND, Brouwer KLR (2010). Sandwich-cultured hepatocytes: an in vitro model to evaluate hepatobiliary transporter-based drug interactions and hepatotoxicity. Drug Metab Rev.

[185] Liu X (1998). Partial maintenance of taurocholate uptake by adult rat hepatocytes cultured in a collagen sandwich configuration. Pharm Res.

[186] Bell CC (2018). Comparison of hepatic 2D sandwich cultures and 3D spheroids for long-term toxicity applications: a multicenter study. Toxicological sciences : an official journal of the Society of Toxicology.

[187] Ruoss M (2018). A standardized collagen-based scaffold improves human hepatocyte shipment and allows metabolic studies over 10 days. Bioengineering (Basel).

[188] Lee J (2009). In vitro toxicity testing of nanoparticles in 3D cell culture. Small.

[189] Haldar S, Lahiri D, Roy P. Chapter 5 - 3D print technology for cell culturing, in 3D printing technology in nanomedicine, N. Ahmad, P. Gopinath, and R. Dutta, Editors. Elsevier. 2019;83–114.

[190] Chang R, et al. Biofabrication of a three-dimensional liver micro-organ as an in vitro drug metabolism model*.* Biofabrication. 2010;2(4):045004. 10.1088/1758-5082/2/4/045004 21079286

[191] Ma X (2016). Deterministically patterned biomimetic human iPSC-derived hepatic model via rapid 3D bioprinting. Proc Natl Acad Sci U S A.

[192] Nguyen AH (2016). MMP-mediated mesenchymal morphogenesis of pluripotent stem cell aggregates stimulated by gelatin methacrylate microparticle incorporation. Biomaterials.

[193] Nguyen DG, et al. Bioprinted 3D primary liver tissues allow assessment of organ-level response to clinical drug induced toxicity in vitro*.* PLoS One. 2016;11(7):e0158674. 10.1371/journal.pone.0158674 PMC4936711 27387377

[194] Pampaloni F, Stelzer E (2010). Three-dimensional cell cultures in toxicology. Biotechnol Genet Eng Rev.

[195] Wong SF (2011). Concave microwell based size-controllable hepatosphere as a three-dimensional liver tissue model. Biomaterials.

[196] Friedrich J (2009). Spheroid-based drug screen: considerations and practical approach. Nat Protoc.

[197] Foty R (2011). A simple hanging drop cell culture protocol for generation of 3D spheroids. Journal of visualized experiments : JoVE.

[198] Achilli T-M, Meyer J, Morgan JR (2012). Advances in the formation, use and understanding of multi-cellular spheroids. Expert Opin Biol Ther.

[199] Otsuka H (2013). Micropatterned co-culture of hepatocyte spheroids layered on non-parenchymal cells to understand heterotypic cellular interactions. Sci Technol Adv Mater.

[200] Edmondson R (2014). Three-dimensional cell culture systems and their applications in drug discovery and cell-based biosensors. Assay Drug Dev Technol.

[201] Chang TT, Hughes-Fulford M (2009). Monolayer and spheroid culture of human liver hepatocellular carcinoma cell line cells demonstrate distinct global gene expression patterns and functional phenotypes. Tissue Eng Part A.

[202] Fey SJ, Wrzesinski K (2012). Determination of drug toxicity using 3D spheroids constructed from an immortal human hepatocyte cell line. Toxicol Sci.

[203] Vorrink SU (2018). Prediction of drug-induced hepatotoxicity using long-term stable primary hepatic 3D spheroid cultures in chemically defined conditions. Toxicol Sci.

[204] Messner S (2013). Multi-cell type human liver microtissues for hepatotoxicity testing. Arch Toxicol.

[205] Tostoes RM (2012). Human liver cell spheroids in extended perfusion bioreactor culture for repeated-dose drug testing. Hepatology.

[206] Peshwa MV (1996). Mechanistics of formation and ultrastructural evaluation of hepatocyte spheroids. In Vitro Cell Dev Biol Anim.

[207] Riccalton-Banks L (2003). Long-term culture of functional liver tissue: three-dimensional coculture of primary hepatocytes and stellate cells. Tissue Eng.

[208] Ramaiahgari SC (2014). A 3D in vitro model of differentiated HepG2 cell spheroids with improved liver-like properties for repeated dose high-throughput toxicity studies. Arch Toxicol.

[209] Elje E, et al. Hepato(geno)toxicity assessment of nanoparticles in a HepG2 liver spheroid model*.* Nanomaterials (Basel). 2020;10(3). 10.3390/nano10030545 PMC7153628 32197356

[210] Dubiak-Szepietowska M (2016). Development of complex-shaped liver multicellular spheroids as a human-based model for nanoparticle toxicity assessment in vitro. Toxicol Appl Pharmacol.

[211] Mikhail AS, Eetezadi S, Allen C. Multicellular tumor spheroids for evaluation of cytotoxicity and tumor growth inhibitory effects of nanomedicines in vitro: a comparison of docetaxel-loaded block copolymer micelles and Taxotere(R)*.* PLoS One. 2013;8(4):e62630. 10.1371/journal.pone.0062630 PMC3633836 23626842

[212] Huch M (2015). Long-term culture of genome-stable bipotent stem cells from adult human liver. Cell.

[213] Prior N, Inacio P, Huch M (2019). Liver organoids: from basic research to therapeutic applications. Gut.

[214] Akbari S (2019). Next-generation liver medicine using organoid models. Front Cell Dev Biol.

[215] Palazzolo S, et al. An effective multi-stage liposomal DNA origami nanosystem for in vivo cancer therapy*.* Cancers (Basel). 2019;11(12). 10.3390/cancers11121997 PMC6966502 31842277

[216] de Graaf IA (2010). Preparation and incubation of precision-cut liver and intestinal slices for application in drug metabolism and toxicity studies. Nat Protoc.

[217] Palma E, Doornebal EJ, Chokshi S (2019). Precision-cut liver slices: a versatile tool to advance liver research. Hepatol Int.

[218] Wu X (2018). Precision-cut human liver slice cultures as an immunological platform. J Immunol Methods.

[219] Palma E, Doornebal EJ, Chokshi S (2019). Precision-cut liver slices: a versatile tool to advance liver research. Hep Intl.

[220] Olinga P (2001). Rat liver slices as a tool to study LPS-induced inflammatory response in the liver. J Hepatol.

[221] Dragoni S, et al. Gold nanoparticles uptake and cytotoxicity assessed on rat liver precision-cut slices*.* toxicological Sciences. 2012;128(1):186–197. 10.1093/toxsci/kfs150 22539612

[222] Hui AY, Friedman SL (2003). Molecular basis of hepatic fibrosis. Expert Rev Mol Med.

[223] Bartucci R, et al. Time-resolved quantification of nanoparticle uptake, distribution, and impact in precision-cut liver slices*.* Small. 2020;16(21):1906523. 10.1002/smll.201906523 32077626

[224] van Midwoud PM (2010). A microfluidic approach for in vitro assessment of interorgan interactions in drug metabolism using intestinal and liver slices. Lab Chip.

[225] Vaira V (2010). Preclinical model of organotypic culture for pharmacodynamic profiling of human tumors. Proc Natl Acad Sci.

[226] Hattersley SM, Greenman J, Haswell SJ (2011). Study of ethanol induced toxicity in liver explants using microfluidic devices. Biomed Microdevices.

[227] Freeman AE, Hoffman RM (1986). In vivo-like growth of human tumors in vitro. Proc Natl Acad Sci U S A.

[228] Vaira V (2010). Preclinical model of organotypic culture for pharmacodynamic profiling of human tumors. Proc Natl Acad Sci U S A.

[229] Piera T (2016). Organ culture model of liver for the study of cancer treatment for hepatocellular carcinoma. Cancer Res J.

[230] Nath S, Devi GR (2016). Three-dimensional culture systems in cancer research: focus on tumor spheroid model. Pharmacol Ther.

[231] Hassan S (2020). Liver-on-a-chip models of fatty liver disease. Hepatology.

[232] Toh YC (2009). A microfluidic 3D hepatocyte chip for drug toxicity testing. Lab Chip.

[233] Filippi C (2004). Improvement of C3A cell metabolism for usage in bioartificial liver support systems. J Hepatol.

[234] Gebhardt R, Mecke D (1979). Perifused monolayer cultures of rat hepatocytes as an improved in vitro system for studies on ureogenesis. Exp Cell Res.

[235] Bhatia SN, Ingber DE (2014). Microfluidic organs-on-chips. Nat Biotechnol.

[236] Gerlach JC (2003). Use of primary human liver cells originating from discarded grafts in a bioreactor for liver support therapy and the prospects of culturing adult liver stem cells in bioreactors: a morphologic study. Transplantation.

[237] Li L (2019). A microfluidic 3D hepatocyte chip for hepatotoxicity testing of nanoparticles. Nanomedicine.

[238] Lu RXZ, Radisic M (2021). Organ-on-a-chip platforms for evaluation of environmental nanoparticle toxicity. Bioactive Materials.

[239] Bhise NS (2014). Organ-on-a-chip platforms for studying drug delivery systems. J Control Release.

[240] Gebhardt R (2003). New hepatocyte in vitro systems for drug metabolism: metabolic capacity and recommendations for application in basic research and drug development, standard operation procedures. Drug Metab Rev.

[241] Li L (2019). A microfluidic 3D hepatocyte chip for hepatotoxicity testing of nanoparticles. Nanomedicine (Lond).

[242] Liu Y, Wang S, Wang Y. Patterned fibers embedded microfluidic chips based on PLA and PDMS for Ag nanoparticle safety testing*.* Polymers (Basel). 2016;8(11). 10.3390/polym8110402 PMC6431932 30974676

[243] Wikswo JP. The relevance and potential roles of microphysiological systems in biology and medicine*.* Exp biol medicine (Maywood, N.J.). 2014;239(9):1061–1072. 10.1177/1535370214542068 PMC4330974 25187571

[244] Ashammakhi N (2020). Microphysiological systems: next generation systems for assessing toxicity and therapeutic effects of nanomaterials.

[245] Zhang YS, Zhang Y-N, Zhang W (2017). Cancer-on-a-chip systems at the frontier of nanomedicine. Drug Discovery Today.

[246] Lee SA (2013). Spheroid-based three-dimensional liver-on-a-chip to investigate hepatocyte-hepatic stellate cell interactions and flow effects. Lab Chip.

[247] Louhimies S (2002). Directive 86/609/EEC on the protection of animals used for experimental and other scientific purposes. Altern Lab Anim.

[248] Neff EP (2017). Printing cures: organovo advances with 3D-printed liver tissue. Lab Anim (NY).

[249] Tolikas M, Antoniou A, Ingber DE (2017). The Wyss Institute: a new model for medical technology innovation and translation across the academic-industrial interface. Bioeng Transl Med.

[250] Conway GE, et al. Adaptation of the in vitro micronucleus assay for genotoxicity testing using 3D liver models supporting longer-term exposure durations*.* Mutagenesis. 2020. 10.1093/mutage/geaa018 PMC7486679 32780103

[251] Au - Llewellyn SV, et al. Advanced 3D liver models for in vitro genotoxicity testing following long-term nanomaterial exposure*.* JoVE. 2020(160):e61141. 10.3791/61141 32568251

[252] Kermanizadeh A (2019). The importance of inter-individual Kupffer cell variability in the governance of hepatic toxicity in a 3D primary human liver microtissue model. Sci Rep.

[253] Barosova H, et al. An in vitro lung system to assess the proinflammatory hazard of carbon nanotube aerosols*.* Int J Mol Sci. 2020;21(15). 10.3390/ijms21155335 PMC7432093 32727099

[254] Au - Braakhuis HM, et al. An air-liquid interface bronchial epithelial model for realistic, repeated inhalation exposure to airborne particles for toxicity testing*.* JoVE. 2020(159):e61210. 10.3791/61210 32478724

[255] Au - Barosova H, et al. Multicellular human alveolar model composed of epithelial cells and primary immune cells for hazard assessment*.* JoVE. 2020(159):e61090. 10.3791/61090 32449722

[256] Ude VC (2019). Using 3D gastrointestinal tract in vitro models with microfold cells and mucus secreting ability to assess the hazard of copper oxide nanomaterials. J Nanobiotechnology.

[257] Segovia-Miranda F (2019). Three-dimensional spatially resolved geometrical and functional models of human liver tissue reveal new aspects of NAFLD progression. Nat Med.

[258] Goulart E, et al. 3D bioprinting of liver spheroids derived from human induced pluripotent stem cells sustain liver function and viability in vitro*.* Biofabrication. 2019;12(1):015010. 10.1088/1758-5090/ab4a30 31577996

[259] Yang H, et al. Three-dimensional bioprinted hepatorganoids prolong survival of mice with liver failure*.* Gut. 2020;gutjnl-2019–319960. 10.1136/gutjnl-2019-319960 PMC7873413 32434830

[260] Wang L (2016). Automated quantitative assessment of three-dimensional bioprinted hydrogel scaffolds using optical coherence tomography. Biomed Opt Express.

[261] Kizawa H (2017). Scaffold-free 3D bio-printed human liver tissue stably maintains metabolic functions useful for drug discovery. Biochemistry and Biophysics Reports.

[262] NCATS. https://ncats.nih.gov/pubs/features/3d-bioprinting. 2018; Available from: https://ncats.nih.gov/pubs/features/3d-bioprinting.

[263] Livingston CA, Fabre KM, Tagle DA (2016). Facilitating the commercialization and use of organ platforms generated by the microphysiological systems (Tissue Chip) program through public–private partnerships. Comput Struct Biotechnol J.

[264] Kaiser J. Seven years later, NIH center that aims to speed drugs to market faces challenges. 2019 [cited 2022 04/01]; Available from: https://www.sciencemag.org/news/2019/09/seven-years-later-nih-center-aims-speed-drugs-market-faces-challenges.

[265] Zhang YS (2017). 3D bioprinting for tissue and organ fabrication. Ann Biomed Eng.

[266] Campbell KH (1996). Sheep cloned by nuclear transfer from a cultured cell line. Nature.

[267] Yue F (2014). A comparative encyclopedia of DNA elements in the mouse genome. Nature.

[268] Brownell L. A swifter way towards 3D-printed organs. 2019 [cited 2022 04/01]; Available from: https://wyss.harvard.edu/news/a-swifter-way-towards-3d-printed-organs/.

[269] Huh D (2010). Reconstituting organ-level lung functions on a chip. Science.

[270] Herland A (2020). Quantitative prediction of human pharmacokinetic responses to drugs via fluidically coupled vascularized organ chips. Nature Biomedical Engineering.

